# Phytochemical profiling, antioxidant and anti-inflammatory potential of methanolic extracts of *Moringa oleifera* (L.) Lam. and *Moringa stenopetala* (Bak.) Cufod. leaves grown in Arba Minch, Ethiopia

**DOI:** 10.1039/d5ra05914c

**Published:** 2025-11-12

**Authors:** Masresha Ahmed Assaye, Marinella De Leo, Duccio Volterrani, Hagos Tesfay, Frehiwot Teka, Eyob Debebe, Solomon Genet Gebre

**Affiliations:** a Department of Internal Medicine, School of Medicine, College of Health Sciences, Addis Ababa University Addis Ababa Ethiopia masresha.ahmed@aau.edu.et hagos.tesfay@aau.edu.et; b Department of Medical Biochemistry, School of Medicine, College of Health Sciences, Addis Ababa University Addis Ababa Ethiopia solgen73@yahoo.com; c Department of Pharmacy, University of Pisa 56126 Pisa Italy marinella.deleo@unipi.it; d Interdepartmental Research Center Nutrafood, “Nutraceuticals and Food for Health”, University of Pisa 56124 Pisa Italy; e CISUP, Centre for Instrumentation Sharing, University of Pisa 56127 Pisa Italy; f Department of Translational Research and of New Technologies in Medicine and Surgery, University of Pisa 56126 Pisa Italy duccio.volterrani@unipi.it; g Traditional and Modern Medicine Research and Development Directorate, Armauer Hansen Research Institute Addis Ababa Ethiopia tekafrehiwot05@gmail.com wisdom.eyob@gmail.com

## Abstract

The genus *Moringa* contains 14 species, including the widely cultivated *Moringa oleifera* and *Moringa stenopetala*. They have outstanding nutritional and medicinal advantages. This study aimed to analyze and compare the anti-inflammatory potential, antioxidant capacity, and phytochemical profile of the two species' methanolic leaf extracts. The ultra-high-performance liquid chromatography-high resolution mass spectrometry method was utilized for phytochemical profiling. Total flavonoid content and phenolic content were determined using the colorimetric procedures of aluminum chloride and Folin–Ciocalteu assays, respectively. *In vitro*, antioxidant activity was measured using 2,2′-casino-bis(3-ethylbenzothiazoline-6-sulfonic acid) and 2,2-diphenyl-1-picrylhydrazyl assays. Protein denaturation and protease activity inhibition assays were employed to determine the anti-inflammatory effects. *M. oleifera* and *M. stenopetala* showed similar chemical profiles, with 29 compounds tentatively identified, including glucosinolates, hydroxycinnamic acids, and flavonoid glycosides. *M. stenopetala* had significantly higher (*p* < 0.05) total phenolic content (74.23 ± 2.65 mg GAE per g) than that of *M. oleifera* (66.33 ± 0.40 mg GAE per g), and *M. stenopetala* also had a greater total flavonoid content (11.27 ± 0.48 mg CE per g) compared to *M. oleifera* leaf extract (9.19 ± 0.06 mg CE per g) (*p* < 0.05). *M. stenopetala* had better antioxidant activity than *M. oleifera*. Both species of *Moringa* suppressed egg albumin denaturation and protease activity. *M. stenopetala* inhibits protein denaturation and protease activity more effectively than *M. oleifera*. In conclusion, this study showed that both plant species have antioxidant and anti-inflammatory properties, with *M. stenopetela* exhibiting greater activity than *M. oleifera*.

## Abstract 2

Phytochemical profiles, antioxidant, and anti-inflammatory activities were assessed in methanolic extracts of *Moringa oleifera* (L.) Lam. and *Moringa stenopetala* (Bak.) Cufod. leaves, using a workflow from plant collection to lab assays.

## Graphical abstract

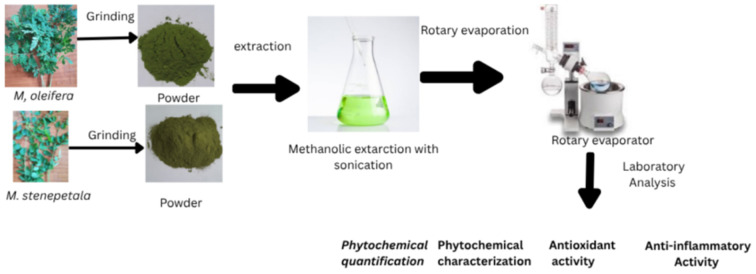

## 1. Introduction

Inflammation is a sequence of biological responses consisting of processes such as protein denaturation, vascular permeability, and reorganization in the structure of membranes.^1^ Oxidative stress occurs when the generation of pro-oxidant molecules exceeds the ability of an organism to neutralize them. This discrepancy could be attributed to augmented oxidant generation and/or impaired antioxidant defenses, contributing significantly to initiating and maintaining the inflammation, which is essential to the pathogenesis of diseases like diabetes, neurodegeneration, metabolic syndrome, and cancer.^2^ Reactive nitrogen and oxygen species production and activity are both significantly reduced by antioxidants.^3^

Researchers are looking into plant-derived compounds for the creation of new drug entities because they have been shown to have a variety of therapeutic benefits.^4,5^ Throughout history, plant-derived natural products have been used as therapeutic agents, with many ancient civilizations using plant-based foods as medicine.^6^ Phytochemicals are the various compounds that greatly influence human health due to their numerous physiological impacts.^7^ The therapeutic values of plants depend on their chemical constituents, the phytochemicals.^8^ Inflammatory conditions are often treated with drugs. However, these agents are to blame for some serious side effects: the biggest culprits being GI irritation and ulcer formation.^9^ Thus, in an effort to find bioactive compounds with antioxidant and anti-inflammatory qualities that have therapeutic potential while reducing side effects, researchers have been looking more and more at medicinal plants.^10,11^

The genus *Moringa* contains 14 species, including the widely cultivated *M. oleifera* Lam. and *M. stenopetala* (Baker f.) Cufod^12,13^ known for their good nutritional and therapeutic properties.^14^ The most frequently studied is *M. oleifera*,^15^ which is cultivated in various nations across the globe, such as South and Central America, Africa, Southeast Asia, and India.^16^*M. stenopetala*, also known as the African *Moringa* tree, is commonly planted in southern Ethiopia, eastern Somalia, and northern Kenya. In Ethiopia, this plant goes by many local names: Haleko in Derashe, Shelagta in Konso, Halako in Gofa and Wolaita, and Shiferaw in Amharic.^17,18^ It is a vegetable source for about 5 million people in southern Ethiopia. The Konso people grow this tree in large areas around their home gardens and farms for both food and medicine. In the Gamo Gofa and Wolaita regions, people traditionally cook and eat the leaves of this plant alongside their *kurkufa* dish, which is made from maize and sorghum grains.^17,19^*M. stenopetala* is widely used in traditional medicine for treating different illnesses, leading to significant research interest in it.^15^

*Moringa* species have gained global attention due to their rich phytochemical diversity and broad spectrum of bioactivities. Recent studies have revealed that *Moringa* species contain diverse phytochemicals, including flavonoids, terpenoids, tannins, anthocyanins, and proanthocyanidins, which contribute to their antioxidant, anti-inflammatory, antimicrobial, anticancer, and cardiovascular health benefits.^18,20–22^ However, changes in a plant's origin or variety can lead to different metabolite phenotypes because these factors affect the makeup of the plant's metabolites.^23^ The amount and composition of the bioactive compounds in a plant affect its pharmacological characteristics. Important influences on the productivity and quality of plants are the kind of temperature, soil, type of water, and light intensity. Plants of the same species grown in different environments might also vary substantially in the amount of bioactive compounds they contain.^24,25^ The phytochemical compositions have shown variability across different countries, and there is limited research on Ethiopian-grown *M. stenopetala* and *M. oleifera*. Furthermore, we employed an optimized extraction approach (methanol + sonication + centrifugation) combined with UHPLC-HR-ESI-Orbitrap/MS technology to analyze both species cultivated at the same location. While extensive literature has investigated the biological activity and chemical content of *M. oleifera*,^26^ fewer studies were performed on *M. stenopetala*.^27^ Beyond phytochemical characterization, we also investigated the *in vitro* anti-inflammatory potential of *M. stenopetala* leaves, which, to our knowledge, has not been previously reported. Therefore, this study takes a new approach by using UHPLC-HR-ESI-Orbitrap/MS technology to analyze the phytochemical profiling of these species. This will help address gaps in knowledge and provide a detailed understanding of the phytochemical composition of these two species in Ethiopia. Additionally, the study analyzes and compares the anti-inflammatory and antioxidant abilities of *M. stenopetala* and well-researched *M. oleifera*.

## 2. Methods and materials

### 2.1. Chemicals

Formic acid, UHPLC-grade, and water were acquired from Deltek (Italy). Aluminum chloride (AlCl_3_), butylated hydroxytoluene (BHT), ascorbic acid, Folin–Ciocalteu's phenol reagent, casein, catechin, potassium persulfate, perchloric acid, sodium nitrite (NaNO_2_), sodium hydroxide (NaOH), Tris–HCl, trypsin., 2,2′-azino-bis(3-ethylbenzothiazoline-6-sulfonic acid) (ABTS), 2,2-diphenyl-1-picrylhydrazyl (DPPH), and gallic acid were acquired from Sigma-Aldrich (USA). Aspirin was purchased from Medtech Ethiopia, which distributes Bayer Aspirin.

### 2.2. Source of *Moringa* leaves

Fresh leaves of the two species were collected from Arba Minch, Ethiopia. Arba Minch is located at 6°01′59″ N and 37°32′59″ E, at an altitude of 1269 m above sea level, receives an average annual rainfall of 952.1 mm, and is 505 km away from the capital city.^28^ Soils are deep, dark in color, and have a texture like clay.^29^ This location was preferred specifically for the availability and abundance of *Moringa* trees in the vicinity. All plant materials were deposited at Addis Ababa University, College of Natural Sciences, National Herbarium, for future reference after a taxonomist confirmed the validity. *M. oleifera* and *M. stenopetala* are represented by the voucher numbers MA001 and MA002, respectively.

### 2.3. Sample collection and preparation

Fresh, mature, and healthy leaves from two *Moringa* species were collected from 14 randomly selected mother trees, each at the optimal maturity stage, typically between 2 to 3 years of age. The composite sample was made by combining leaves from different locations and parts of the trees (*e.g.*, top, middle, and bottom branches) to represent the overall chemical profile of the species. Approximately 150 grams of leaves were collected from each tree, totaling 2 kilograms. The leaves were placed in plastic bags with ventilation holes to prevent moisture buildup during transport and were immediately delivered to the laboratory. Upon arrival, the leaves were sanitized for one and 5 min with 70% ethanol and sodium hypochlorite, respectively, and then repeatedly washed with distilled water and dried in the shade. Powdered in an electric multifunction mill (HYDDNice: Model HY-AB-0326, China), then passed through a screen (20 mesh). Finally, they were placed in a refrigerator at −20 °C for storage.

### 2.4. *Moringa* leaf extracts preparation

Five grams of each powder were extracted with 100 mL of methanol, and then two sonication cycles (40 °C, 60 min, 80%) and centrifugation for 9 min at 3500 rpm were applied (using a Beckman Coulter Allegra 21R refrigerated centrifuge, USA). A Buchi R-210 rotary evaporator (Switzerland) at 40 °C was used to concentrate the filtrates from the two cycles. The obtained extracts were viscous in consistency. The concentrated extracts were kept at −20 °C.^20,30^ Methanol was chosen as the organic solvent for this study because it is more polar than other types of alcohol, like ethanol, acetone, and others. In many cases, methanol extraction gave the best results. Especially for extracting leaves in various situations using sonication.^30^ The safety of *M. oleifera's* methanolic extract at low and moderate concentrations was validated by a cytotoxicity study that was published in the literature.^21^ This supports both the efficiency and relative safety of the chosen extraction method. The extraction yield was calculated as shown in eqn (1),^31^1
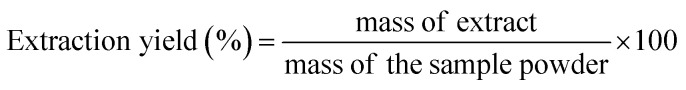


### 2.5. Chemical characterization of the extracts by LC-HR-Orbitrap/ESI-MS

The chemical investigation of both *M. oleifera* and *M. stenopetala* methanolic leaf extracts was performed using a Vanquish Flex UHPLC system equipped with a diode array detector and a high-resolution mass spectrometry (HR-MS) Q Exactive Plus Orbitrap-based FT-MS, which was equipped with an electrospray ionization (ESI) source (Thermo Fisher Scientific Inc., Bremen, Germany). Methanol (MeOH) was used to dissolve the extracts and create mixtures containing 1 mg mL^−1^. A 5 µL supernatant was obtained by centrifuging the mixture for 10 minutes at 2710×*g*. Then, the mixture was introduced into an LC-MS system that was fitted with a SecurityGuard™ Ultra Cartridge (Phenomenex, Bologna, Italy) and a C18 Kinetex® Biphenyl column (100 × 2.1 mm, 2.6 µm particle size), provided by a SecurityGuard™ Ultra Cartridge (Phenomenex, Italy). The chromatography was performed with a linear solvent gradient, increasing solvent B from 5% to 60% over 20 minutes, with a flow rate of 0.5 mL min^−1^. The mobile phase consisted of solvent A (0.1 v/v formic acid in water) and solvent B (0.1 v/v formic acid in methanol). The column's temperature was maintained at 4 °C.

ESI-HR mass spectra were obtained using both negative and positive ESI modes in the *m*/*z* 135–2000 range. For full MS studies, the resolution was set to 70 000, with a maximum injection time of 220 ms. For data-dependent MS/MS scans, the resolution was set at 17 500, and the maximum injection time was 60 ms. The ionization parameters were optimized according to previously described methods.^32^ UV spectra were recorded between 200–600 nm, and three specific channels were selected at 254, 280, and 325 nm.

### 2.6. Total phenolic content (TPC)

To measure TPC Folin–Ciocalteu method^33^ was slightly modified. One mL of 10-fold diluted Folin–Ciocalteu reagent was added to 0.1 mL of the extract (1 mg mL^−1^). Then, the mixture was allowed to stand for 5 minutes before 1 mL (7.5% w/w) of sodium carbonate was added. After incubating for 90 minutes at room temperature, the absorbance was measured at 765 nm. The gallic acid calibration curve equation (*y* = 0.0083*x* − 0.0622, *R*^2^ = 0.980) was used to express the data as mg of gallic acid equivalent per gram of dry extract (mg GAE per g).

### 2.7. Total flavonoid content (TFC)

The aluminum chloride colorimetric method^33^ was slightly modified to measure TFC. After diluting the extracts (1 mg mL^−1^) with 1.25 mL of distilled water, 0.75 µL of 5% NaNO_2_ was added. After 6 minutes, 150 µL of 10% AlCl_3_ was added, followed by 1 mL of NaOH after another 5 minutes. The resulting pink color was immediately measured at 510 nm. Standard solutions of catechin at varying concentrations were prepared to construct a calibration curve (*y* = 0.0583*x* − 0.0494, *R*^2^ = 0.978), and the results were expressed in milligrams of catechin equivalents per gram of dry extract (mg CE per g).

### 2.8. Antioxidant activity

#### 2.8.1. DPPH (2,2-diphenyl-1-picrylhydrazyl radical) radical-scavenger assay

With a few minor adjustments, the DPPH radical-scavenging method^34^ was used to determine the antioxidant activity of the extract. 50 to 1000 µg mL^−1^ of extracts were used. For every mL of extract, 2 mL of DPPH solution (0.06% w/v) was added and left to remain at room temperature in the dark for 30 min. Methanol (100%) was used as a blank, and ascorbic acid and BHT were used as positive controls. At 520 nm, the solutions' absorbance was measured. Percent DPPH radical scavenging capability calculated using eqn (2).2
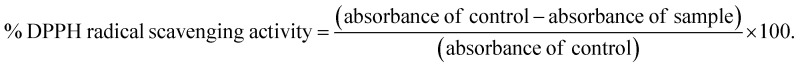


#### 2.8.2. ABTS (2,2′-azino-bis(3-ethylbenzothiazoline-6-sulphonic)) radical-scavenger assay

Using a slightly modified version of,^35^ the ABTS radical-scavenger assay was determined. Various extract concentrations (50 to 1000 µg mL^−1^) were used. The ABTS radical cation (ABTS^+^) was created by mixing 2.45 mM potassium persulfate (pH 7.4) and a 7 mM ABTS stock solution. The mixture was incubated in the dark for 12 hours before use. The resultant ABTS^+^ solution was diluted with ethanol to reach an absorbance of 0.700 ± 0.02 at 734 nm. Thereafter, 200 µL of each sample and 4 mL of ABTS^+^ solution were combined, incubated for 15 minutes, and at 734 nm, the absorbance was measured. The two positive controls were BHT and ascorbic acid. Methanol served as the blank. Eqn (3) was used to calculate the percentage ABTS radical scavenging capability.3
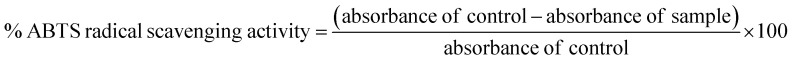


### 2.9. Anti-inflammatory activity

#### 2.9.1. Inhibition of protein denaturation

The study assessed the capacity of *M. oleifera* and *M. stenopetala* leaf extract to prevent albumin denaturation using the methods described by,^36,37^ with slight modification. A reaction mixture (5 mL) was made by using 2.8 mL of phosphate buffer (pH 6.4), 0.2 mL of fresh hen's egg albumin, and 2 mL of extract (100–500 µg mL^−1^) concentration. The concentration range applied in this assay differs from that used in the DPPH assay, as it was specifically selected based on preliminary optimization studies, solubility constraints, and previously reported literature values, to ensure accurate assessment of anti-inflammatory activity. Positive and negative controls were aspirin and deionized water, respectively. After 20 minutes of incubation at 37 °C, test solutions were heated to 70 °C for five minutes. After cooling, the samples were measured at 660 nm, and eqn (4) was used to determine the percentage inhibition.4
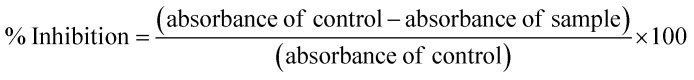


#### 2.9.2. Protease inhibition activity

With a few minor adjustments, the proteinase inhibitory activity method^37,38^ was utilized to evaluate the extract's proteinase inhibitory activity. A reaction mixture (2 mL) was made using 0.06 mg of trypsin, 1 mL of Tris–HCl buffer (20 mM, pH 7.4), and 1 mL of test extract (100–500 µg mL^−1^). After 5 min of incubation at 37 °C, 1 mL of casein (0.8% w/v) was added to the mixture. A second 20 minute incubation period was applied to the mixture. The process was subsequently halted by adding 2 mL of a 70% v/v perchloric acid solution. To obtain the supernatant, it was centrifuged for 5 min at 5000 rpm, and the absorbance was measured at 210 nm. Tris–HCl buffer was used as a blank. A solution of Tris–HCl and aspirin was used as the negative and positive control, respectively. Percentage inhibition was calculated using eqn (5).5
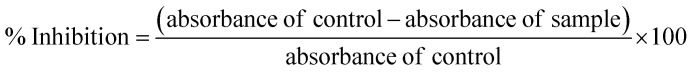


### 2.10. Data analysis

With Xcalibur 4.1 software (Thermo Fisher Scientific Inc., Germany), all MS data were processed. The IC_50_ values were estimated by nonlinear curve-fitting. The Student's *t*-test was utilized to compare the differences between the two groups, and one-way ANOVA with Tukey's post hoc test was utilized to assess group differences. The GraphPad Prism version 10.2.3 was used for analysis. Each experimental measurement was conducted thrice, and findings were reported as average ± standard deviation (SD). *p* < 0.05 was considered statistically significant.

## 3. Results

The percentage extraction yield of both species was calculated. The percentage extraction yield of *Moringa oleifera* and *Moringa stenopetala* was 24.5% and 24.7%, respectively.

### 3.1. Chemical fingerprint of *Moringa* extracts

**Fig. 1:**
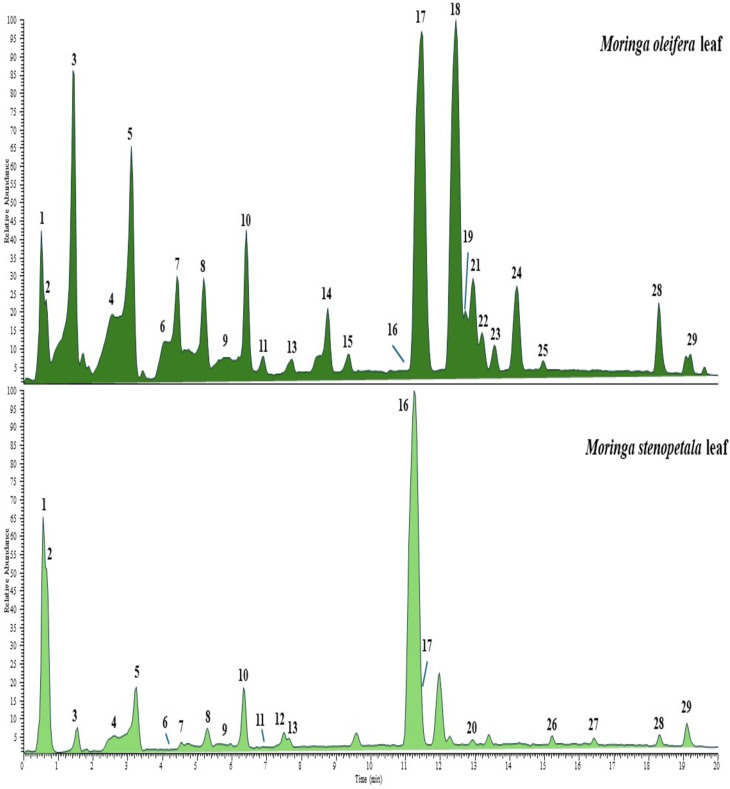
UHPLC-HR-ESI-MS/Orbitrap profiles, registered in negative ionization mode, of methanol extracts of *Moringa oleifera* and *Moringa stenopetala* leaves. Peak data are shown in Table 1.

Fig. 1 displays the chromatograms that were obtained operating in negative ion mode. The components of the two *Moringa* species were tentatively identified by comparing their elution order, molecular formula, full MS, and MS/MS data (Table 1) with literature data,^39–42^ considering an accepted mass error <5 ppm. Fragmentation mass spectra of all compounds are provided in Fig. S1–S11. As a result, 29 compounds were tentatively characterized as glucosinolates, hydroxycinnamic acids, and flavonoid glycosides. Results showed that the two extracts shared many components, differing in the presence of several compounds and their level of production. Glucosinolates are compounds well known as constituents of *Moringa* plants. According to the literature,^40^ two isomers of glucomoringin (1 and 3, [M–H]^−^ at *m*/*z* 570.0960), a hydroxy-aromatic glucosinolate disaccharide, were found in both extracts. Furthermore, compound 10 ([M–H]^−^ at *m*/*z* 612.1066) was also annotated as a glucosinolate, as deduced by the existence of product ions at *m*/*z* 96.06 (HSO_4_^−^) and 79.96 (HSO_3_^−^) in the MS/MS experiments (Fig. S1). Due to the possibility of different isomeric forms, compound 10 could be attributed to 4-(2′-*O*-acetyl-α-l-rhamnopyranosyloxy) benzyl glucosinolate or 4-(3′-*O*-acetyl-α-l-rhamnopyranosyloxy) benzyl glucosinolate or 4-(4′-*O*-acetyl-α-l-rhamnopyranosyloxy) benzyl glucosinolate.^39^

**Table 1:** UHPLC-HR-ESI-Orbitrap/MS and chromatographic (*t*_R_ = retention time) data registered in negative ionization mode of compounds detected in the methanol extracts of *Moringa oleifera* (MO) and *Moringa stenopetala* (MS) leaves

No.^a^	Compound^b^	*t*_R_ (min)	[M–H]^−^ (*m*/*z*)	Product ions^c^ (*m*/*z*)	Formula	Error	Extract
1	Glucomoringin I	0.51	570.0960	259.01, **96.06**, 79.96	C_20_H_29_NO_14_S_2_	+0.579	MO, MS
2	Citric acid	0.69	191.0194	173.00, **111.01**, 87.00	C_6_H_8_O_7_	+1.728	MO, MS
3	Glucomoringin II	1.42	570.0960	259.01, **96.06**, 79.96	C_20_H_29_NO_14_S_2_	−0.579	MO, MS
4	Caffeoylquinic acid I (3-caffeoylquinic acid)	2.54	353.0880	**191.06**, 173.04, 179.03, 135.04	C_16_H_18_O_9_	−0.538	MO, MS
5	Caffeoylquinic acid II (5-caffeoylquinic acid)	3.11	353.0880	**191.06**, 173.04, 179.03, 135.04	C_16_H_18_O_9_	−0.538	MO, MS
6	*p*-Coumaroylquinic acid I (3-*p*-coumaroylquinic acid)	4.07	337.0932	191.06, **163.04**, 119.05	C_16_H_18_O_8_	−0.920	MO, MS
7	*p*-Coumaroylquinic acid II (5-*p*-coumaroylquinic acid)	4.42	337.0932	191.06, **163.04**, 119.05	C_16_H_18_O_8_	−0.920	MO, MS
8	Caffeoylquinic acid III (4-*p*-caffeoylquinic acid)	5.19	353.0880	191.06, **173.04**, 179.03, 135.04	C_16_H_18_O_9_	−0.538	MO, MS
9	Feruloylquinic acid	6.19	367.1035	**193.05**, 173.04, 149.06	C_17_H_20_O_9_	−0.109	MO, MS
10	4-(2′-*O*-Acetyl-α-*l*-rhamnopyranosyloxy)benzyl glucosinolate or 4-(3′-*O*-acetyl-α-*l*-rhamnopyranosyloxy)benzyl glucosinolate or 4-(4′-*O*-acetyl-α-*l*-rhamnopyranosyloxy) benzyl glucosinolate	6.42	612.1066	370.10, 259.01, **96.96**, 79.96	C_22_H_31_NO_15_S_2_	−0.588	MO, MS
11	*p*-Coumaroylquinic acid III (4-*p*-coumaroylquinic acid)	6.88	337.0932	191.06, **173.04**, 119.05	C_16_H_18_O_8_	−0.920	MO, MS
12	Roseoside	7.49	431.1924 [M + HCOO]^−^	**385.19**, 223.13, 153.09	C_19_H_30_O_8_	−0.301	MS
13	Benzyl alcohol xylopyranosyl-(1,6)-glucopyranoside (benzyl b-primeveroside)	7.65	447.1511 [M + HCOO]^−^	401.43, **269.10**, 161.05	C_18_H_26_O_10_	−0.671	MO, MS
14	Apigenin 6,8-*C*-glucoside (vicenin 2)	8.77	593.1519	503.12, 473.11, 383.08, **353.07**	C_27_H_30_O_15_	−1.197	MO, MS
15	Alkyl b-primeveroside	9.34	439.1824 [M + HCOO]^−^	**393.18**, 191.06, 179.06, 161.04, 149.05	C_17_H_30_O_10_	−0.683	MO
16	Rutin	11.23	609.1464	301.04, **300.03**, 271.02, 255.03	C_30_H_26_O_14_	−0.476	MS
17	Quercetin glucoside	11.46	463.0886	301.04, **300.03**, 271.02, 255.03	C_21_H_20_O_12_	−0.864	MO, MS
18	Quercetin malonylhexoside I (quercetin 3-*O*-(6ʺ-malonylglucoside))	12.45	549.0888	505.10, 463.09, **300.03**, 301.04, 271.02	C_24_H_22_O_15_	−0.382	MO, MS
19	Quercetin 3-hydroxy-3-methylglutarylglucoside	12.72	607.1302	545.13, 505.10, 463.09, 301.04, **300.03**, 271.02, 255.03	C_27_H_28_O_16_	+0.428	MO
20	Kaempferol rutinoside	12.93	593.1517	284.03, **285.04**, 255.03, 227.03	C_27_H_30_O_15_	−0.860	MS
21	Kaempferol glucoside	12.95	447.0936	**284.03**, 285.04, 255.03, 227.03	C_21_H_20_O_11_	−0.693	MO, MS
22	Quercetin malonylhexoside II	13.21	549.0888	505.10, 463.09, **300.03**, 301.04, 271.02	C_24_H_22_O_15_	−0.382	MO, MS
23	Isorhamnetin glucoside	13.56	477.1041	**314.04**, 315.05, 299.02, 285.04, 271.02	C_22_H_22_O_12_	−0.524	MO
24	Kaempferol malonylhexoside	14.21	533.0939	489.10, 284.03, **285.04**, 267.03, 255.03	C_24_H_22_O_14_	−0.413	MO, MS
25	Isorhamnetin malonylhexoside	14.98	563.1044	519.11, 314.04, **315.05**, 299.01	C_25_H_24_O_15_	−0.284	MO
26	Sebacic acid	15.20	201.1129	183.10, 157.12, 139.11, 111.08	C_10_H_18_O_4_	+1.641	MS
27	Jasmonic acid	16.42	209.1181	180.97, **59.01**	C_12_H_18_O_3_	+1.052	MS
28	Trihydroxyoctadecadienoic acid	18.29	327.2178	309.21, 291.20, 229.14, 211.13	C_18_H_32_O_5_	−0.306	MO, MS
29	Trihydroxyoctadecenoic acid	19.22	329.2335	311.22, 293.21, 211.13	C_18_H_34_O_5_	−0.456	MO, MS

a Peak numbers correspond to those of Fig. 1 and are listed in elution order.

b Compounds were tentatively identified by comparison of mass spectra (full scan and fragmentation pathway) with literature data.

c The base ion peak is shown in bold.

Hydroxycinnamic acids (**4–9**, 11) were represented by caffeoylquinic, *p*-coumaroylquinic, and feruloylquinic acids found as isomers in both extracts, with *M. oleifera* having the highest content compared to *M. stenopetala* leaves. Hydroxycinnamoyl quinic acids are known to be produced in plants as positional isomers since the esterification can occur at C3, C4, and C5 hydroxides, and occasionally at C1.^43^ Furthermore, *trans* isomers are generally produced in plants, while exposure of extracts to UV light or the MS electric field can generate *cis* isomers.^44^ Based only on MS data, it is difficult to discriminate against *Regio*-isomers since common product ions in the ESI-MS/MS experiments were generated. However, based on the elution order reported in a previous study by Masike *et al.*^43^ positional isomers were suggested in Table 1. The identification of 4-caffeoylquinic acid can be supported by the presence of a base ion peak at *m*/*z* 173.04, instead of 191.06 as observed in the isomers 4 and 5 (ref. 45) (Fig. S2). Among flavonols, glycosides of quercetin (**16–19**, and 22), kaempferol (20, 21, and 24), and isorhamnetin (23 and 25) were found, but differently distributed in the two plants. Rutin (16) was the prominent peak detected in *M. stenopetala*, present only in traces in *M. oleifera*; on the contrary, quercetin glucoside (17) was found to be very abundant in *M. oleifera*, together with a quercetin malonylhexoside I (18) tentatively identified as quercetin 3-*O*-(6ʺ-malonylglucoside) previously reported by Amaglo *et al.*^45^ Compound 22 was a quercetin malonylglucoside isomer, probably having a malonyl residue attached to a different position on the glucose moiety. In addition, the other flavonoids were detected only in *M. oleifera*, except for kaempferol rutinoside (20), found as a component only of *M. stenopetala*, even in a low amount. Both extracts showed the presence of a flavone *C*-glycoside, identified as vicenin 2 (or apigenin 6,8-*C*-glucoside).^40^ Finally, minor peaks corresponding to trihydroxy octadecadienoic acids (28 and 29) were detected in both *Moringa* samples.

### 3.2. Total flavonoid and phenolic contents

*M. stenopetala* had significantly higher TPC (74.23 ± 2.65 mg GAE per g) than *M. oleifera* (66.33 ± 0.40 mg GAE per g), as well as a significantly higher TFC (11.27 ± 0.48 mg CE per g) compared to *M. oleifera* (9.19 ± 0.06 mg CE per g) (*p* < 0.05) (Table 2).

**Table 2:** Antioxidant activities, TPC, and TFC of the two *Moringa* species and positive controls^a^

Samples	IC_50_ DPPH (µg mL^−1^)	IC_50_ ABTS (µg mL^−1^)	TPC (mg GAE per g)	TFC (mg CE per g)
*M. oleifera*	57.12 ± 0.09^a^	37.58 ± 0.87^a^	66.33 ± 0.40^b^	9.19 ± 0.06^b^
*M. stenopetala*	44.00 ± 1.61^b^	25.64 ± 1.06^b^	74.23 ± 2.65^a^	11.28 ± 0.48^a^
Ascorbic acid	15.20 ± 0.81^d^	13.42 ± 0.27^d^	Nt	Nt
BHT	24.82 ± 0.93^c^	18.17 ± 0.28^c^	Nt	Nt

a Note: values = mean ± SD (*n* = 3). Different superscript letters (a–d) in the same column indicate statistically significant differences, assessed using one-way ANOVA and Tukey's multiple comparison tests at a *p*-value <0.05. “Nt” indicates that a particular value was not tested.

### 3.3. Antioxidant activity

#### 3.3.1. DPPH radical-scavenging assay

Table 2 shows the IC_50_ values, which were 57.12 ± 0.09 µg mL^−1^ for *M. oleifera*, 44.00 ± 1.61 µg mL^−1^ for *M. stenopetala*, 15.20 ± 0.81 µg mL^−1^ for ascorbic acid, and 24.82 ± 0.93 µg mL^−1^ for butylated hydroxytoluene (BHT). *M. stenopetala* demonstrated superior DPPH radical scavenging activity compared to *M. oleifera*, as evidenced by its lower IC_50_ value (*p* < 0.05). This indicates that *M. stenopetala* required a lower concentration to achieve 50% inhibition of the DPPH radical, suggesting its greater antioxidant potency. Overall, the observed order of DPPH radical scavenging activity was ascorbic acid > BHT > *M. stenopetala* > *M. oleifera*.

#### 3.3.2. ABTS radical-scavenging assay

The IC_50_ values for *M. oleifera*, *M. stenopetala*, ascorbic acid, and BHT were 37.58 ± 0.87, 25.64 ± 1.06, 13.42 ± 0.27, and 18.18 ± 0.28 µg mL^−1^, respectively. *M. stenopetala* has significantly higher scavenging activity than *M. oleifera* (*p* < 0.05). Overall, ABTS scavenging activity was ranked as follows: ascorbic acid > BHT > *M. stenopetala* > *M. oleifera* (Table 2).

### 3.4. Anti-inflammatory activity

#### 3.4.1. Inhibition of protein denaturation

**Fig. 2:**
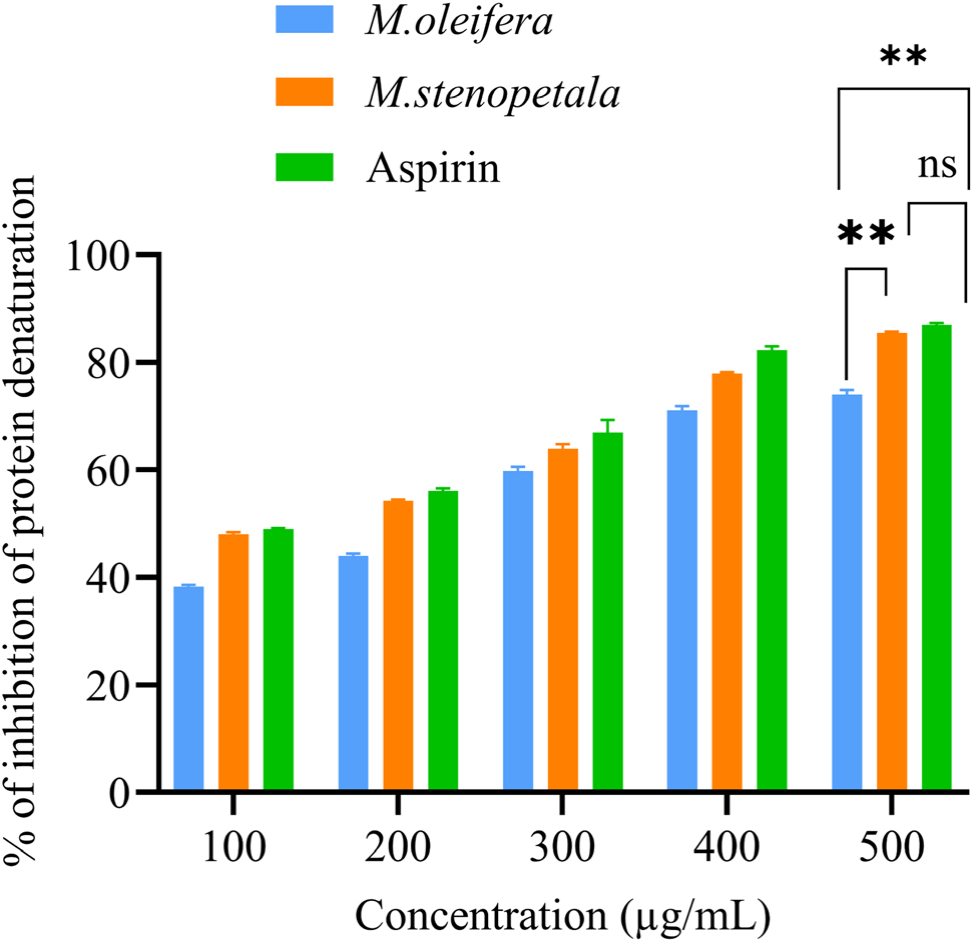
Inhibition of protein denaturation by two *Moringa* species and a positive control at different concentrations. The results were examined using one-way ANOVA and Tukey's multiple comparison test to assess group differences. Any differences **p < 0.01, *p < 0.05 were statistically significant and ns *p* > 0.05: non-signific. (ns).

Fig. 2 summarizes the inhibitory effects of the two *Moringa* species against protein (albumin) denaturation. Both extracts demonstrated a significantly increased dose-dependent inhibition of egg albumin denaturation from 100 to 500 µg mL^−1^ of *M. oleifera*, *M. stenopetala*, and aspirin exhibited maximum inhibition percentages at 500 µg mL^−1^ and a percentage of inhibition of 74.08 ± 0.80, 85.02 ± 0.85, and 89.9 ± 1.37, respectively. *M. stenopetala* and the reference drug, aspirin, showed similar activity with no significant difference between them (*p* > 0.05). However, *M. stenopetala* exhibited a significantly higher inhibition of protein denaturation than *M. oleifera* at concentrations of 500 µg mL^−1^ (*p* < 0.01).

Protein denaturation inhibition was further expressed by IC_50_. Table 3 illustrates that the IC_50_ value for *M. stenopetala* (95.23 ± 1.32 µg mL^−1^) was significantly lower (*p* < 0.05) than that of *M. oleifera* (110.73 ± 2.45 µg mL^−1^) and similar to aspirin (93.01 ± 3.51 µg mL^−1^). This indicates that *M. stenopetala* demonstrates stronger inhibition activity against protein denaturation compared to *M. oleifera* and is equivalent to the reference drug aspirin.

**Table 3:** Anti-inflammatory activities of extracts and positive controls^a^

Samples	Protein denaturation test IC_50_ (µg mL^−1^)	Protease inhibition test IC_50_ (µg mL^−1^)
*M. oleifera*	110.73 ± 2.45^a^	94.56 ± 1.95^a^
*M. stenopetala*	95.23 ± 1.32^b^	91.30 ± 1.70^a^
Aspirin	93.01 ± 3.51^b^	79.15 ± 1.51^b^

a Note: values = mean ± SD (*n* = 3). Different superscript letters (a–b) in the same column indicate statistically significant differences, assessed using one-way ANOVA and Tukey's multiple comparison tests at a *p*-value <0.05.

#### 3.4.2. Protease inhibitory activity

**Fig. 3:**
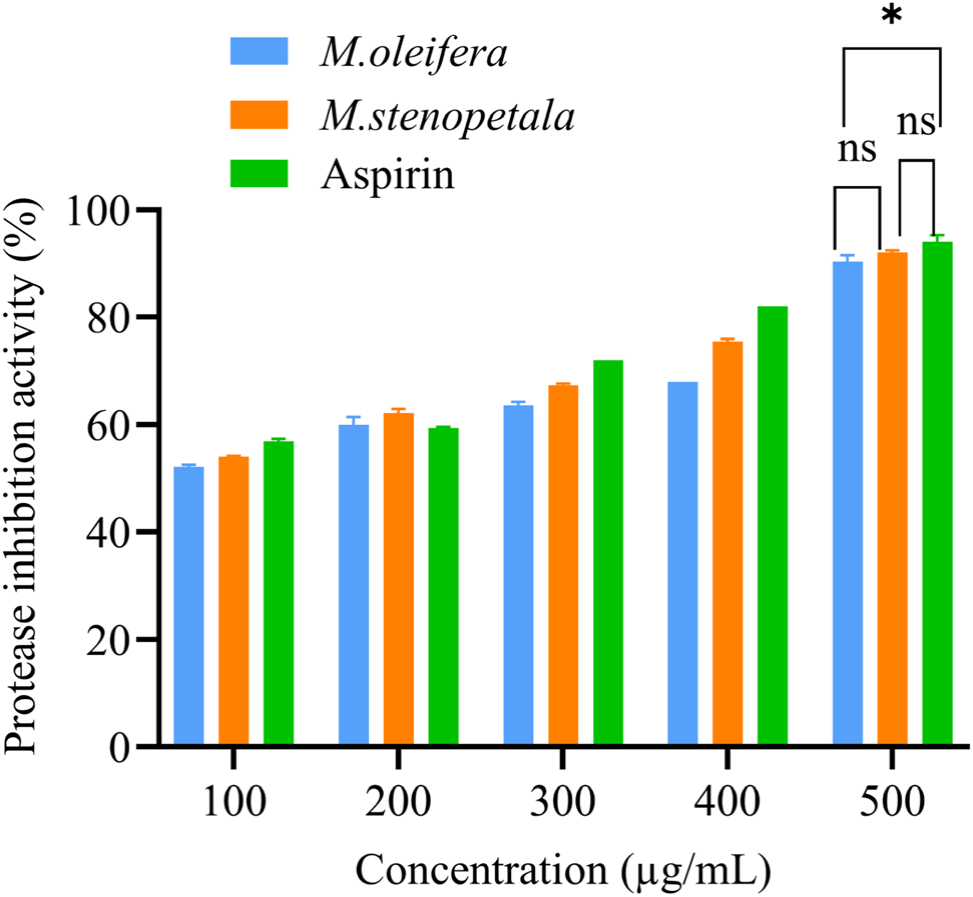
Inhibition of protease by two *Moringa* species and positive control at different concentrations. The results were examined using one-way ANOVA and Tukey's multiple comparison test to assess group differences. Any differences with **p < 0.01, *p < 0.05 were statistically significant and ns *p* > 0.05: non-significant (ns).

Fig. 3 summarizes the inhibitory effects of the two species and a positive control against protease activity. The protease inhibition activity of the test sample and the standard rose linearly in a dose-dependent manner. *M. oleifera*, *M. stenopetala*, and aspirin exhibited maximum inhibition percentages at 500 µg mL^−1^ with percentages of inhibition of 85.10 ± 0.12, 86.98 ± 0.19, and 88.9 ± 0.1%, respectively. The reference drug, aspirin, and the extract from *M. stenopetala* leaf exhibited comparable activity with no discernible differences (*p* > 0.05). Although there was no significant difference between the two species' extracts (*p* > 0.05), aspirin inhibited protease activity more effectively than *M. oleifera* at a concentration of 500 µg mL^−1^.

Both *M. oleifera* and *M. stenopetala* extracts demonstrated significant inhibitory effects against protease activity, 94.56 ± 1.95 µg mL^−1^ and 91.30 ± 1.70 µg mL^−1^ IC_50_ values, respectively. In summary, extracts of the two *Moringa* species effectively inhibit protease activity but exhibit slightly higher IC values compared to aspirin (79.15 ± 1.51 µg mL^−1^), indicating a slightly lower protease inhibitory activity (Table 3).

## 4. Discussion

Our results show that the extracts contain a wide spectrum of bioactive compounds, including glucosinolates, hydroxycinnamic acids, and flavonoid glycosides, all of which have proven medicinal benefits. In the present study, a UHPLC-HR-ESI-MS/Orbitrap system was utilized to fingerprint the chemical constituents in test sample leaf extracts. By comparing with available secondary metabolite databases such as ChemSpider, mzCloud, and available literature, 29 compounds were tentatively characterized. These bioactive compounds' presence in the two species indicates that they could be good sources of bioactive chemicals with considerable health benefits, known for their anti-inflammatory, antioxidant, and possible anticancer activities, and are essential for improving the nutrition and the health of the populations within the region.^46^ Glucosinolates are naturally occurring compounds containing sulfur and nitrogen used for anti-inflammatory, antioxidant, and chemoprotective effects.^47^ Plant-based phenolic compounds called hydroxycinnamic acids (HCAAs) have several health advantages, such as antioxidant, anti-cancer, and anti-inflammatory properties.^48^ Flavonoid glycosides are also known for a range of health benefits. They exhibit significant anti-inflammatory, antioxidant, antiviral, hepatoprotective, anticancer, and antitumor activities.^22,49^ These compounds exhibit dual bioactivities, particularly antioxidant and anti-inflammatory effects. For instance, glucosinolates and their hydrolysis products (*e.g.*, isothiocyanates) can modulate inflammatory pathways by inhibiting pro-inflammatory cytokines and enzymes such as COX-2 and iNOS.^50^ Hydroxycinnamic acids act as radical scavengers while also suppressing NF-κB activation, a key regulator of inflammation.^51^ Similarly, flavonoid glycosides not only neutralize reactive oxygen species (ROS) but also modulate intracellular signaling cascades involved in inflammation, such as MAPK and PI3K/Akt pathways.^21,52^ The multifunctionality of these phytochemicals signifies a mechanistic advantage, supporting their broader therapeutic potential in managing complex disorders characterized by both oxidative and inflammatory stress. The simultaneous presence of glucosinolates, hydroxycinnamic acids, and flavonoid glycosides within the extract also raises the possibility of synergistic interactions, where their combined bioactivities may produce enhanced anti-inflammatory and antioxidant effects compared to isolated compounds.^22,53,54^ This multifunctional and cooperative behavior highlights the therapeutic potential of these phytochemicals.

The extract's TPC and TFC of the two *Moringa* species showed remarkable results from spectrophotometric analysis. The noted phytochemical constituents suggest antioxidant activity and possible health benefits.^55^ This benefit holds value especially for regions where synthetic antioxidants or drugs are not readily available. This makes *Moringa* species ideal candidates for inclusion in contemporary diets or traditional medicine due to their health-promoting properties. We observed that *M. stenopetala* (74.23 ± 2.65 mg GAE per g) had greater TPC (*p* < 0.05) than *M. oleifera* (66.33 ± 0.40 mg GAE per g). This supports the findings of (ref. 16 and 56) where *M. stenopetala* had significantly greater TPC than *M. oleifera*. The TPC values for *M. stenopetala* in this study were higher than those obtained by Dessalegn & Rupasinghe^57^ and Befa *et al.*,^13^ who found the TPC of 39 and 2.6 mg GAE per g, respectively, and lower than those found by Tebeka & Libsu^58^ and Toma *et al.*,^59^ who reported TPC of 92.8 and 79.81 mg GAE per g, respectively. The TPC of the *M. oleifera* in this study exceeded the results of (ref. 60–64) and was lower than the findings of ref. 37 and 65. Variances in the quantities of secondary metabolites, plant maturity age, and genotype could all be contributing causes to the variances in TPC values of *Moringa* leaf extract.^16,66,67^ Our result also showed that the TFC of *M. stenopetala* (11.28 ± 0.48 mg CE per g) was higher than *M. oleifera* (9.19 ± 0.06 mg CE per g). The observation aligns with the,^16^ report, confirming that *M. stenopetala* has significantly greater TFC than *M. oleifera*. Regarding the TFC of *M. stenopetala* detected in this research, it agrees with Befa *et al.*^13^ and Dessalegn & Rupasinghe,^57^ who stated TFC (11.9 ± 0.2 mg CE per g) and (11 ± 2 mg CE per g), respectively, which are lower than those found by Toma *et al.*,^59^ who stated TFC of 71.73 mg CE per g. TFC of *M. oleifera* detected in this research agrees with the previous studies and is higher than the findings of (ref. 60–64) and lower than the findings of ref. 37, 60, 68 and 69. Numerous factors, including genotype, plant age, and varying concentrations of secondary metabolites, could be the cause of the variability in TFC values of *Moringa* leaf extract.^16,66,67^

Plant extracts are categorized by their antioxidant capacity based on their IC_50_ values: extracts with IC_50_ values under 50 µg mL^−1^ are classified as having high antioxidant capacity, those between 50 and 100 µg mL^−1^ have moderate antioxidant capacity, and those over 100 µg mL^−1^ are considered to have low antioxidant capacity.^70,71^ In the present study, *M. stenopetala* had an IC_50_ value of 44.00 ± 1.61 µg mL^−1^ for DPPH radical scavenging activity, which is classified as a strong antioxidant activity, and *M. oleifera* had an IC_50_ value of 57.12 ± 0.09, which is classified as a moderate antioxidant. *M. stenopetala* exhibited considerably better antioxidant capacity than *M. oleifera* (*p* < 0.05). This finding is comparable to that of Ntshambiwa *et al.*^16^*M. stenopetala* showed stronger antioxidant activity (DPPH IC_50_ value of 44.00 ± 1.61 µg mL^−1^), which is higher than the findings stated by Habtemariam^72^ and Desalegn and Rupasinghe,^57^ who reported DPPH IC_50_ values of 59.50 and 78 µg mL^−1^, respectively. *M. oleifera* antioxidant activity (with a DPPH IC_50_ value of 57.12 ± 0.09 µg mL^−1^) observed in the present study agrees with Aziz *et al.*^73^ and Nassrallah *et al.*,^74^ higher than the finding reported by Baldisserotto *et al.*^20^ and Ezz El-Din Ibrahim *et al.*^63^ but lower than the finding stated by Jahan *et al.*,^75^ Prasajak *et al.*,^76^ and Unuigbe *et al.*^77^ Based on the ABTS radical scavenging activity result, *M. stenopetala* had better antioxidant activity than *M. oleifera* in ABTS radical scavenging experiments (*p* < 0.05). Both *Moringa* species demonstrated strong antioxidant activity, likely due to their high phenolic compound content. The antioxidant activity of phenolic compounds is primarily due to their hydroxyl (–OH) groups, which donate hydrogen atoms to neutralize free radicals such as DPPH^+^ and ABTS^+^, resulting in the formation of stable phenoxyl radicals. This stability is enhanced by resonance delocalization and the presence of bulky hydrophobic substituents on the aromatic ring, which further support radical stabilization and improve antioxidant efficiency.^21,78^ Variations in antioxidant capacity primarily stemming from genetic differences between *M. oleifera* and *M. stenopetala*.^16,79^ Environmental factors and secondary metabolites, genetic diversity, plant maturity, and extraction procedures may all contribute to the antioxidant capacity difference between the current research and literature values. Environmental situations can impact variation in active substances and antioxidant activity.^80^ Qadir *et al.*^60^ reported that there was variation in an antioxidant as maturity progressed. Dzięcioł^81^ analyzed how different extraction techniques impact the yield and antioxidant activity. Moreover, temperature, annual precipitation, and soil type from the plant's location also impact the level of antioxidants.^82^

We also identified strong anti-inflammatory effects of the two species. The two species revealed significant inhibitory effects on inflammation. Protein denaturation has been linked to many inflammatory disorders. Therefore, the protein denaturation inhibition assay has been used extensively to evaluate the degree of inhibition exerted by *M. oleifera* and *M. stenopetala* extracts. Thus, the protein denaturation inhibition assay has been utilized as a convenient tool to check the extent of inhibition rendered by the *M. oleifera* and *M. stenopetala* extracts. Our results demonstrate substantial protein denaturation inhibition activity for both *Moringa* species. This suggests that their ability to stabilize protein structures under inflammatory conditions, which is comparable with the findings reported by Saleem *et al.*^37^ and Padmalochana,^36^ who reported that *M. oleifera* extracts strongly inhibited protein denaturation. The IC_50_ value for *M. stenopetala* extract (95.23 ± 1.32 µg mL^−1^) was significantly lower than that of *M. oleifera* (110.73 ± 2.45 µg mL^−1^) and similar to that of aspirin (93.01 ± 3.51 µg mL^−1^). This evidence that *M. stenopetala* could inhibit protein denaturation is better than *M. oleifera* and equals the reference drug aspirin. Similarly, our results demonstrate protease inhibition. Proteases are major players in inflammation, inducing tissue destruction and activation of cytokines.^83^ Our results indicate that extracts of both *Moringa* species effectively inhibit protease activity; however, IC_50_ values were a little bit higher than aspirin, which could mean weaker protease inhibition in this assay. This reveals that the bioactive component of both species is likely to inhibit the enzymatic activities involved in inflammatory processes. Our findings are correlated with other studies, in which Saleem *et al.*^37^ and Padmalochana^36^ found that methanolic extracts of *M. oleifera* had significant protease inhibition capacity. These *Moringa* species' anti-inflammatory properties could be due to their secondary metabolites, like as polyphenols and flavonoids. These chemicals act by blocking pro-inflammatory enzymes, controlling cytokine synthesis, and providing antioxidant action to alleviate oxidative stress and inflammation.^46^

The current study's findings are promising; however, it has some limitations. A key limitation of our study is the restricted range of techniques available in our laboratory, which impacted our ability to fully assess the biological activities of the extracts. Efforts were made to address these limitations through potential collaborations with laboratories abroad. However, further *in vivo* validation, mechanistic studies, and cytotoxicity assessments are still required. To build on our findings and overcome these constraints, we propose the following future research directions: confirm the *in vitro* results with animal models or clinical trials to enhance their biological relevance, pathway analysis or molecular target identification would increase understanding of how these activities are achieved and to further elucidate the mechanisms, molecular modeling of the extracts is necessary to appropriately relate their chemical composition with the biological activities.

## 5. Conclusion

In conclusion, the current study using UHPLC-HR-ESI-Orbitrap/MS revealed the presence of glucosinolates, hydroxycinnamic acids, and flavonoid glycosides in the *M. oleifera* and *M. stenopetala* methanolic leaf extracts. This study also verified that the methanolic leaf extracts of the two species are potentially rich sources of antioxidants and other bioactive compounds. The extract also possesses anti-inflammatory properties, as it has similar effects to aspirin. Notably, this is one of the first reports on the anti-inflammatory activity of *M. stenopetala* leaves. The results highlight *Moringa* species' therapeutic value and have impacts for the development of nutraceuticals that combat oxidative stress and inflammation. These findings also contribute to the standardization of traditional *Moringa*-based medicinal practices. Despite being encouraging, the lack of cytotoxicity and *in vivo* research is a drawback. To gain a better understanding, future work needs to define dosing parameters, toxicity thresholds, and safety profiles for long-term use to increase the practical use of these findings.

## Author contributions

Masresha Ahmed Assaye: conceptualization, methodology, investigation, data curation, formal analysis, writing – original draft, writing – review & editing. Marinella De Leo: methodology, investigation, data curation, formal analysis, writing – review & editing. Duccio Volterrani: conceptualization, methodology, data curation, supervision, writing – review & editing. Hagos Tesfay: conceptualization, methodology, supervision, writing – review & editing. Frehiwot Teka: methodology, investigation, writing – review & editing. Eyob Debebe: methodology, investigation, data curation, writing – review & editing. Solomon Genet Gebre: conceptualization, methodology, supervision, writing – original draft, writing – review & editing.

## Conflicts of interest

The authors declare no conflicts of interest.

## Supplementary Material

RA-015-D5RA05914C-s001

## Data Availability

The data supporting this article have been included in the manuscript and as part of the supplementary information (SI). Supplementary information: the SI provides HR-ESI-MS/MS spectra of all key compounds, calibration curves for determining total phenolic and flavonoid contents, and figures of the studied plant parts. See DOI: https://doi.org/10.1039/d5ra05914c.
