# Supplementary material for: Phytochemical profiling, antioxidant and anti-inflammatory potential of methanolic extracts of Moringa oleifera (L.) Lam. and Moringa stenopetala (Bak.) Cufod. leaves grown in Arba Minch, Ethiopia
Source: RSC Adv. 2025 Nov 12;15(51):43818–29. doi: 10.1039/d5ra05914c (PMC12606576; doi:10.1039/d5ra05914c)
Supplement: RA-015-D5RA05914C-s001 [file RA-015-D5RA05914C-s001.pdf]

Supplementary Information (SI) for RSC Advances.
This journal is © The Royal Society of Chemistry 2025

Supplementary File

Phytochemical Profiling, Antioxidant and Anti-inflammatory Potential of Methanolic Extracts of *Moringa oleifera* (L.) Lam. and *Moringa stenopetala* (Bak.) Cufod. Leaves Grown in Arba Minch, Ethiopia

Masresha Ahmed Assaye,*[ab] Marinella De Leo,[cde] Duccio Volterrani,[f] Hagos Tesfay,[a] Frehiwot Teka,[g] Eyob Debebe[g] and Solomon Genet Gebre*[b]

[a]Department of Internal Medicine, School of Medicine, College of Health Sciences, Addis Ababa University, Addis Ababa, Ethiopia.

[b]Department of Medical Biochemistry, School of Medicine, College of Health Sciences, Addis Ababa University, Addis Ababa, Ethiopia.

[c] Department of Pharmacy, University of Pisa, 56126 Pisa, Italy

[d]Interdepartmental Research Center Nutrafood, "Nutraceuticals and Food for Health," University of Pisa, 56124 Pisa, Italy

[e]CISUP, Centre for Instrumentation Sharing, University of Pisa, 56127 Pisa, Italy

[f]Department of Translational Research and of New Technologies in Medicine and Surgery, University of Pisa, 56126, Pisa, Italy.

[g]Traditional and Modern Medicine Research and Development Directorate, Armauer Hansen Research Institute, Addis Ababa, Ethiopia

* CORRESPONDENCE

Masresha Ahmed Assaye (PhD Candidate)

Email: masresha.ahmed@aau.edu.et ORCID: https://orcid.org/0000-0002-5392-0789

Solomon Genet Gebre (PhD)

Email: solgen73@yahoo.com ORCID: https://orcid.org/0000-0002-5433-0107

Peak 1

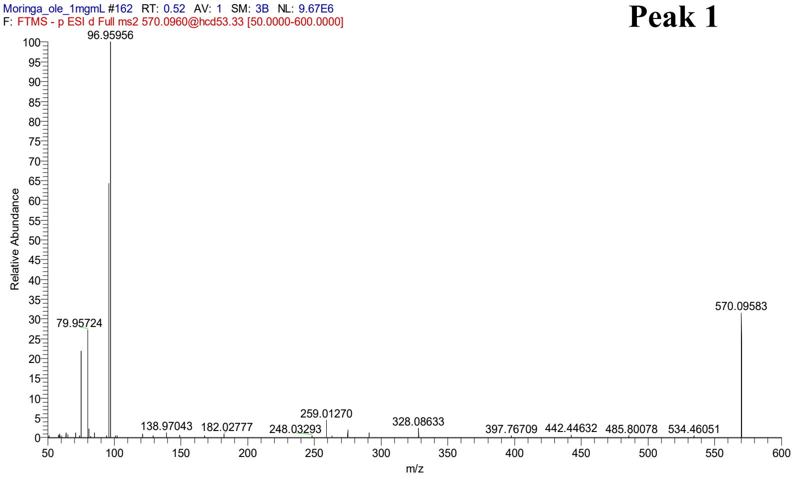

Peak 3

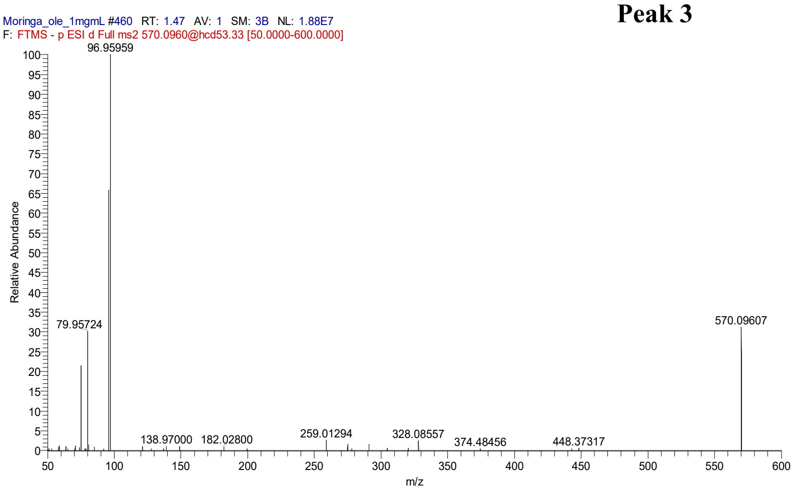

Peak 10

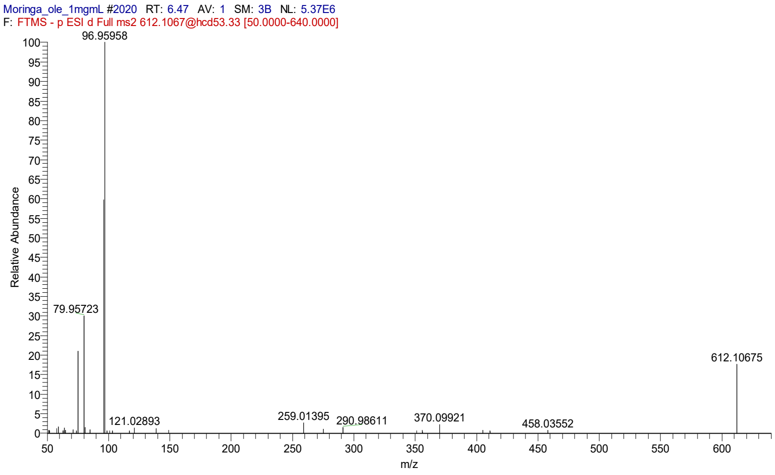

Fig. S1 HR-ESI-MS/MS of glucosinolates (**1**, **3**, and **10**).

.

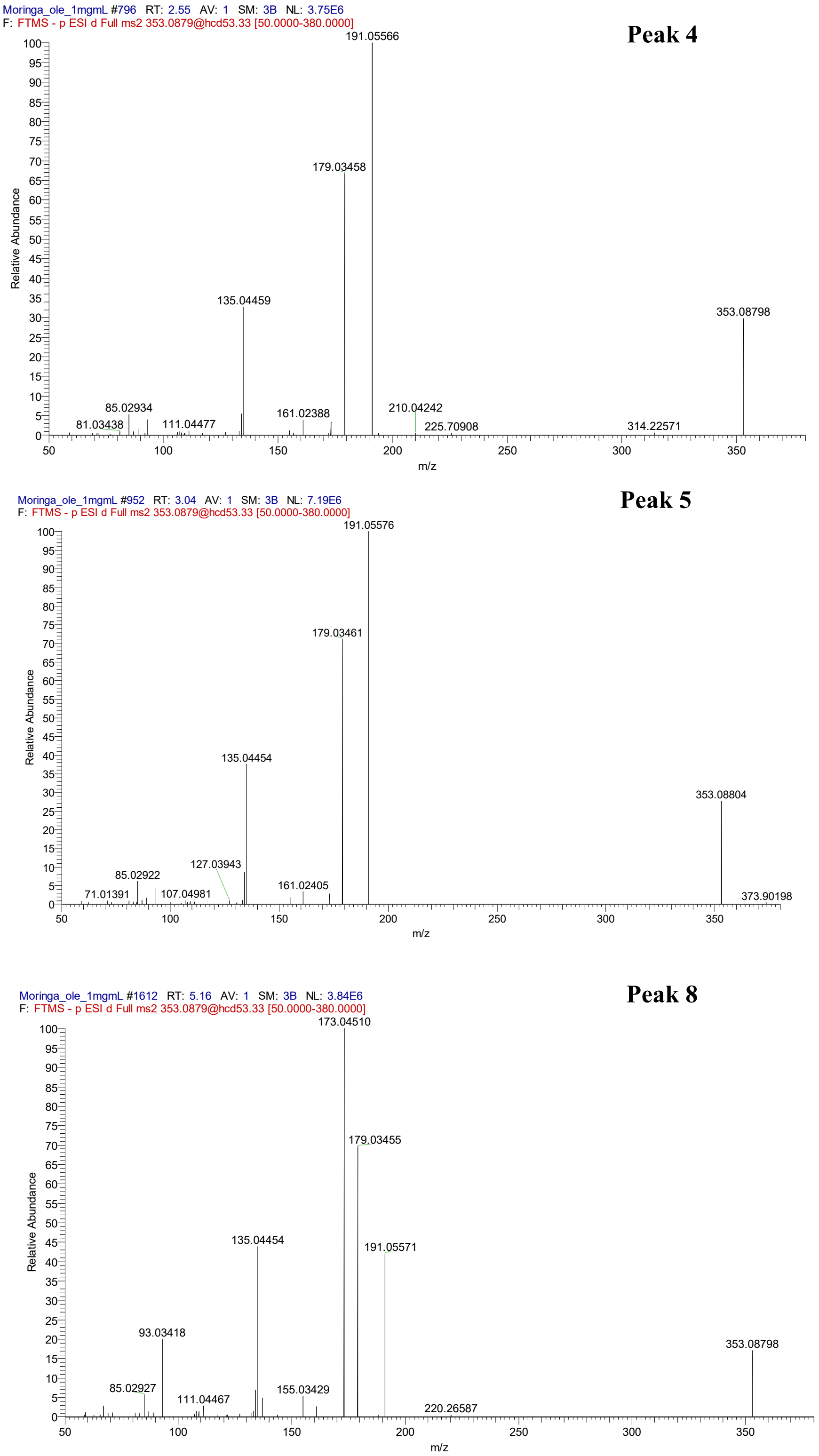

Fig. S2 HR-ESI-MS/MS of hydroxycinnamic acids **4**, **5**, and **8**.

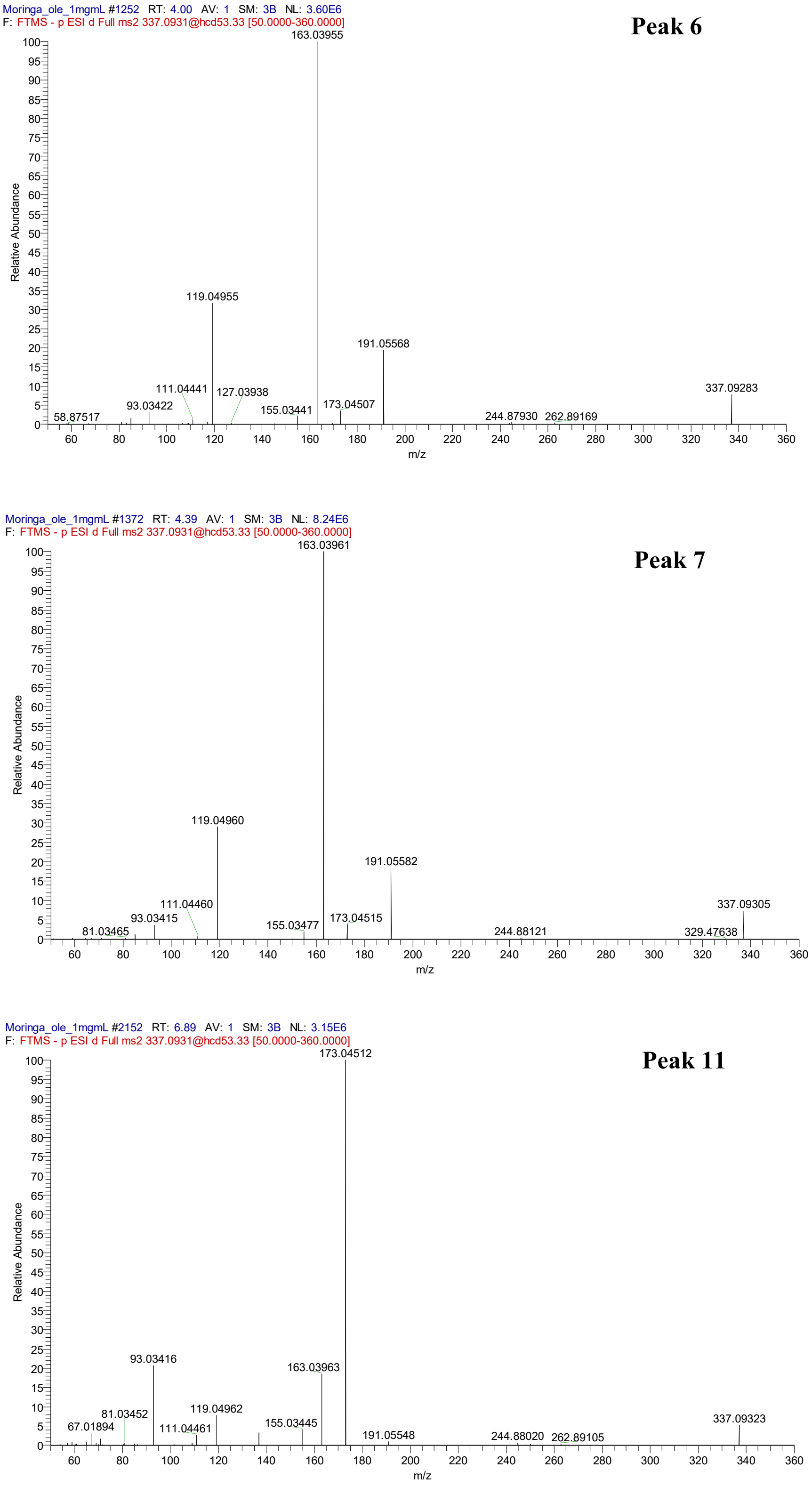

Fig. S3 HR-ESI-MS/MS of hydroxycinnamic acids **6**, **7**, and **11**.

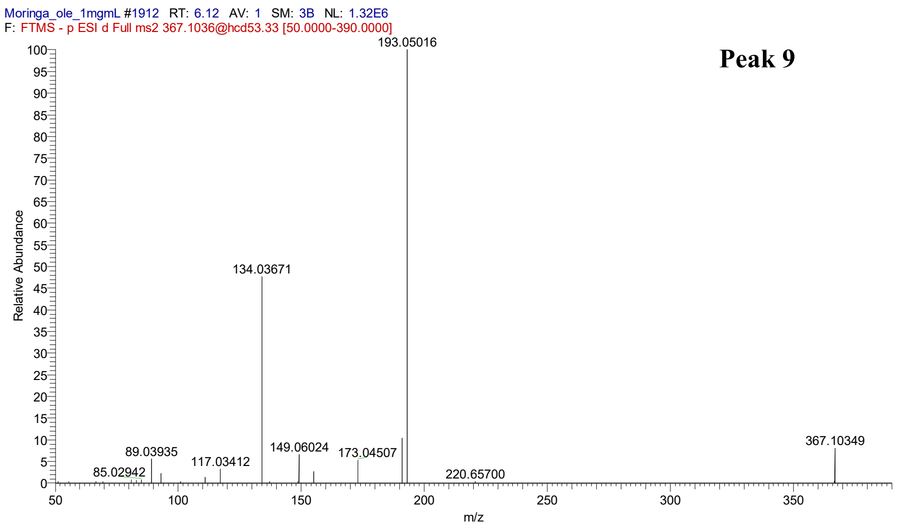

Fig. S4 HR-ESI-MS/MS of hydroxycinnamic acid **9**.

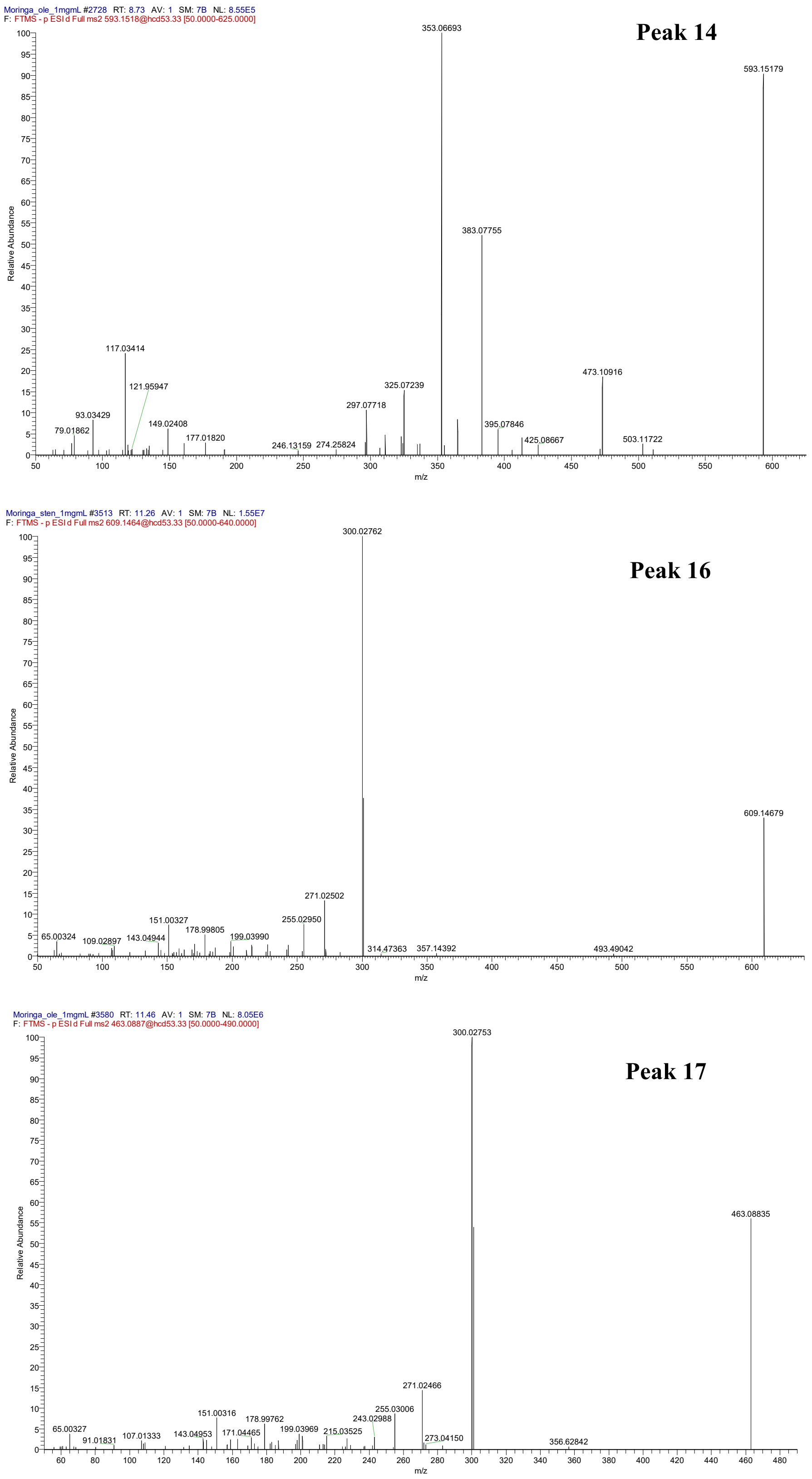

Fig. S5 HR-ESI-MS/MS of flavonoids **14-17**.

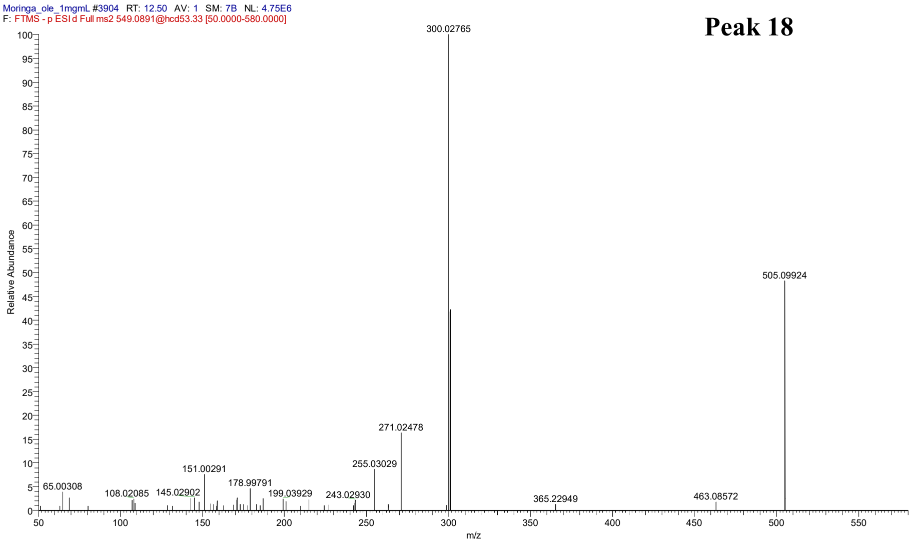

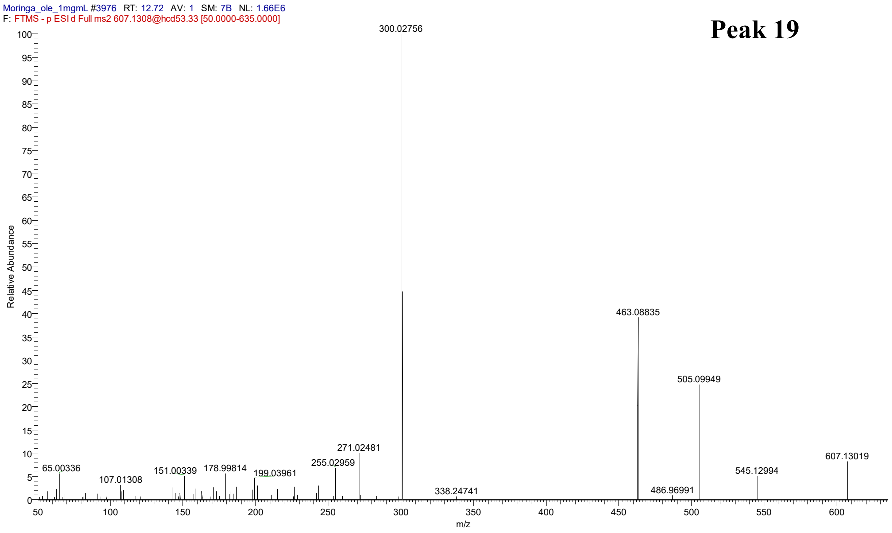

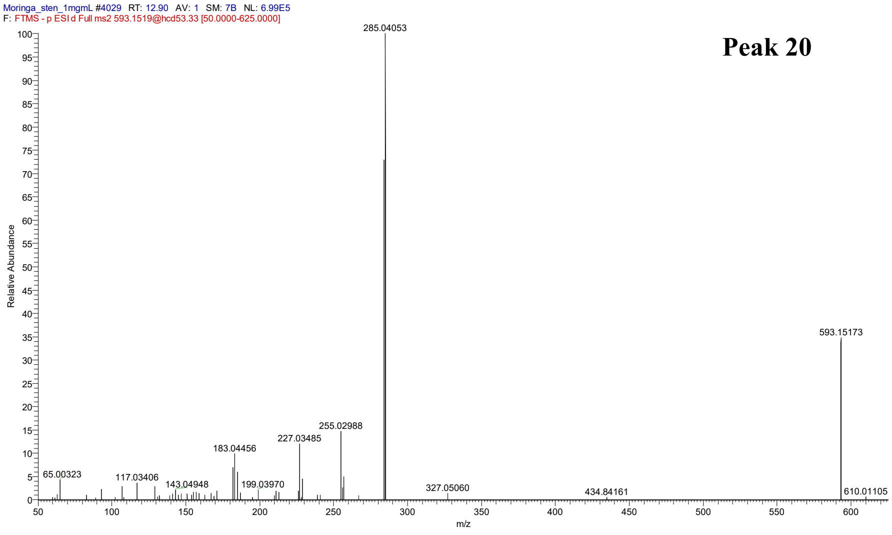

Fig. S6 HR-ESI-MS/MS of flavonoids **18-20**.

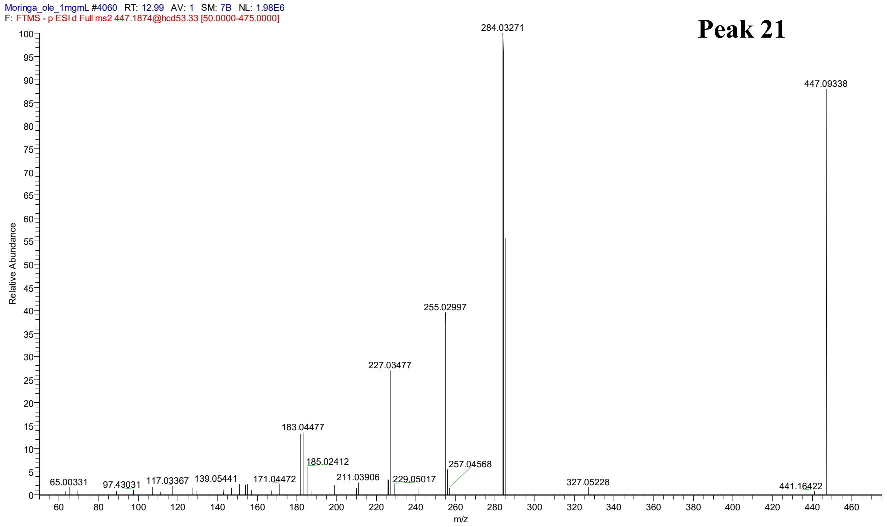

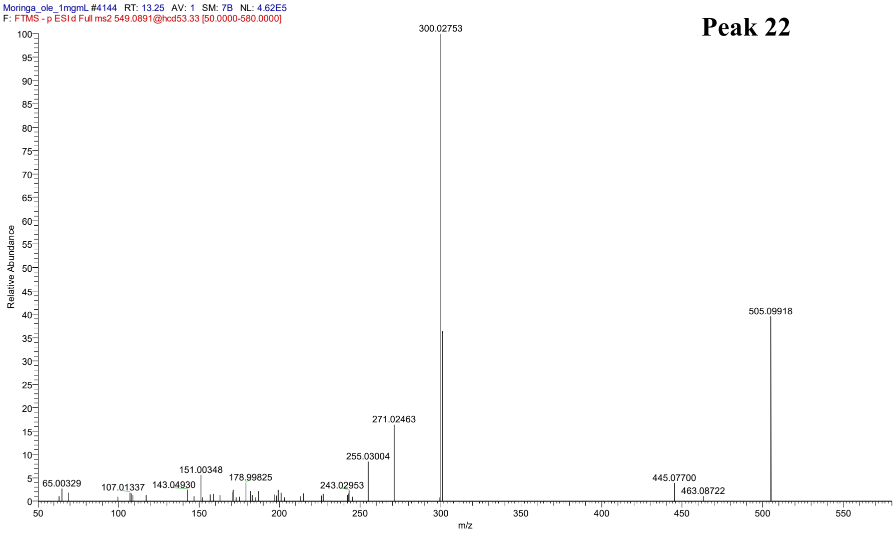

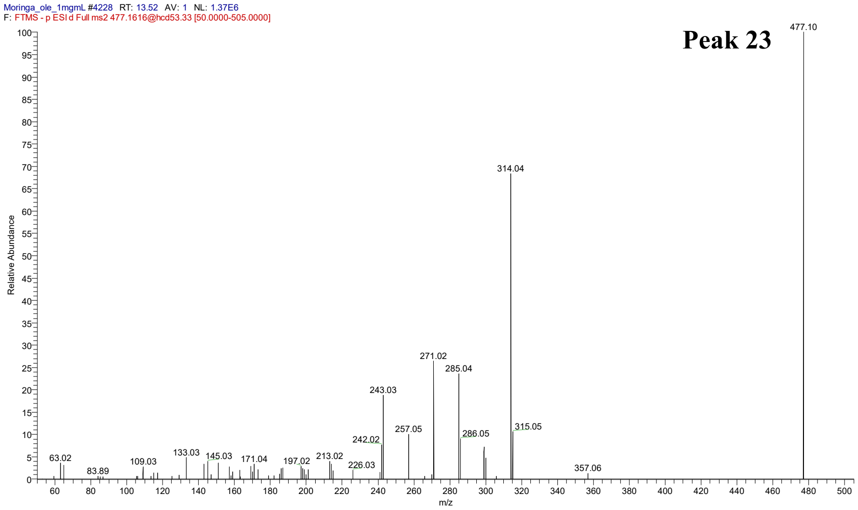

Fig. S7 HR-ESI-MS/MS of flavonoids **21-23**.

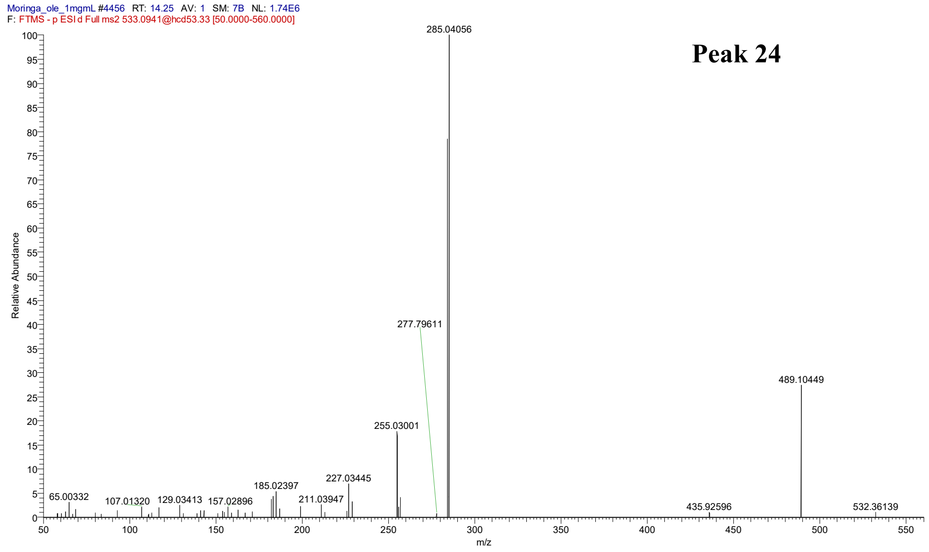

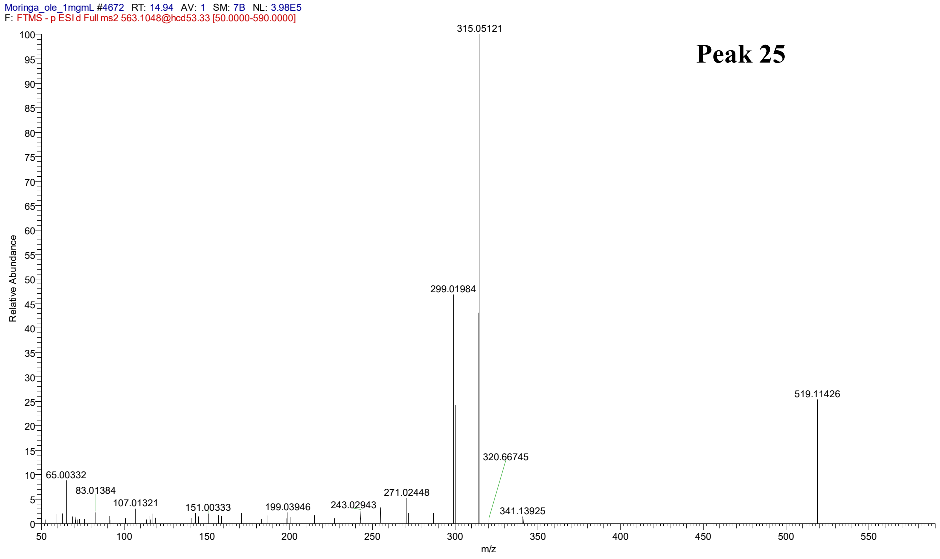

Fig. S8 HR-ESI-MS/MS of flavonoids **24** and **25**.

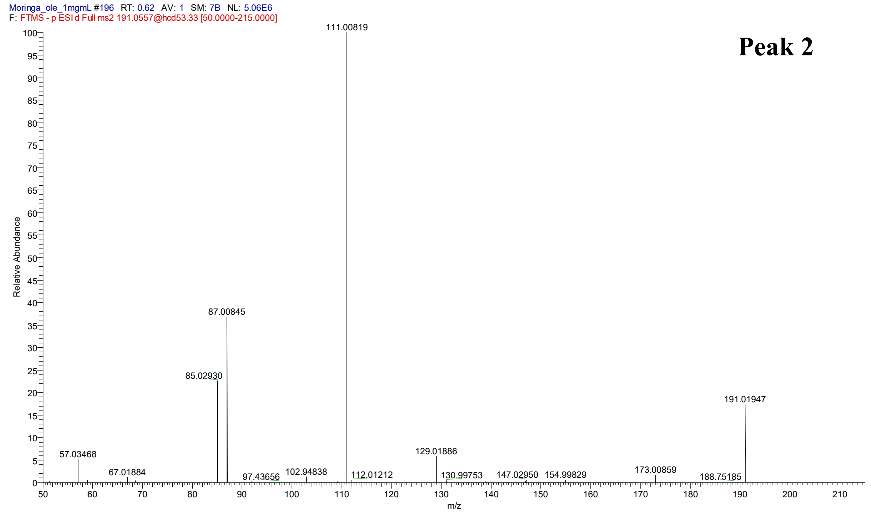

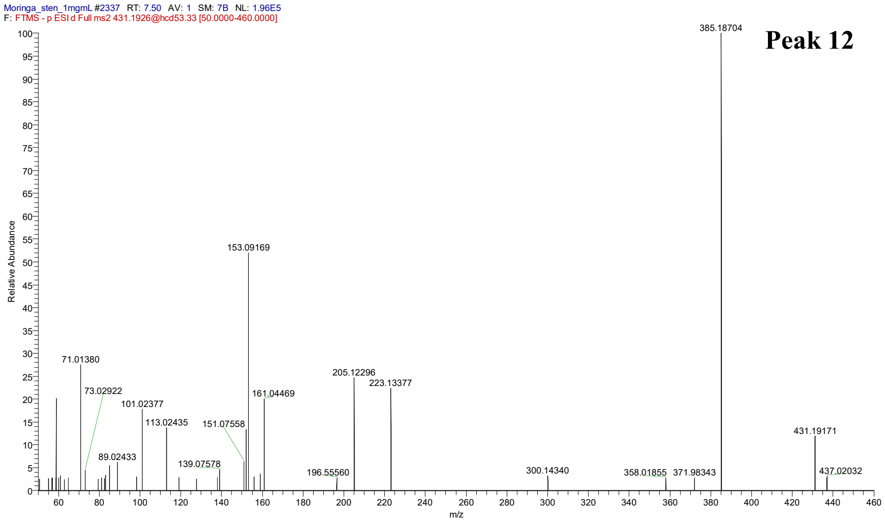

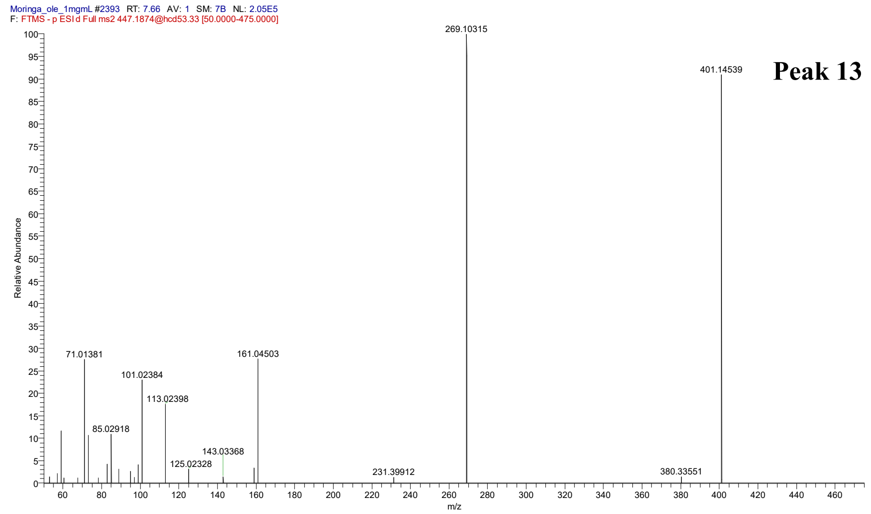

Fig. S9 HR-ESI-MS/MS of peaks **2**, **12**, and **13**.

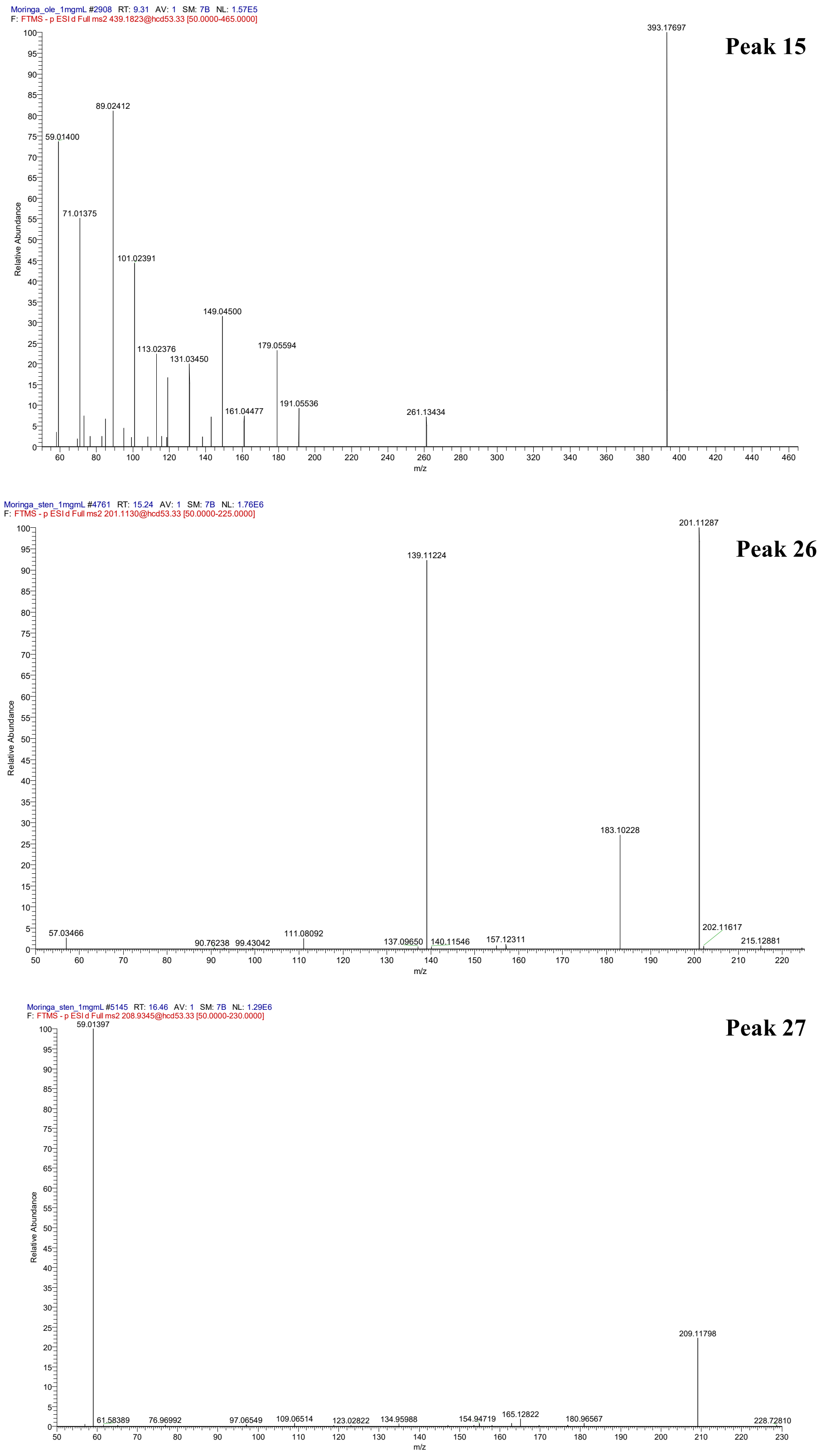

Fig. S10 HR-ESI-MS/MS of peaks **15**, **26**, and **27**.

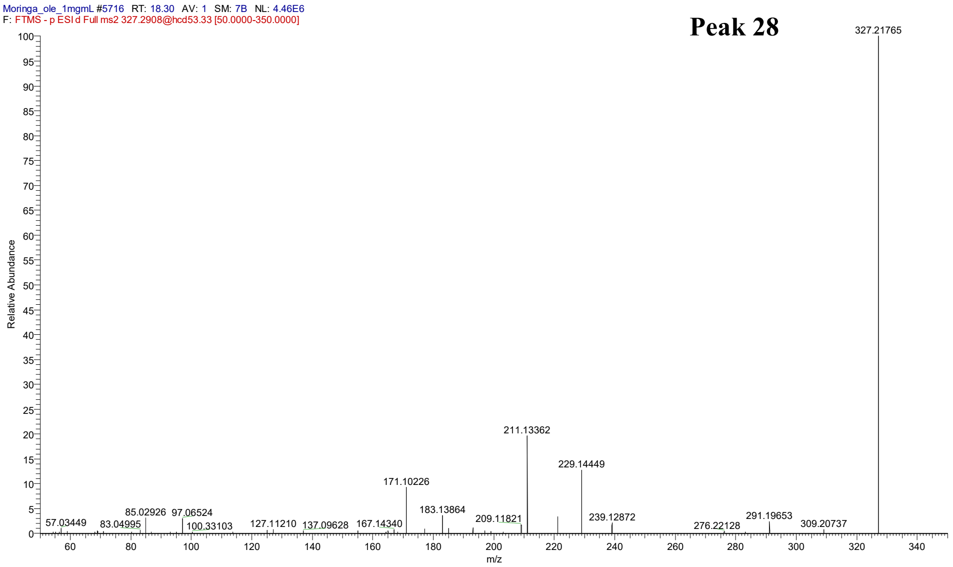

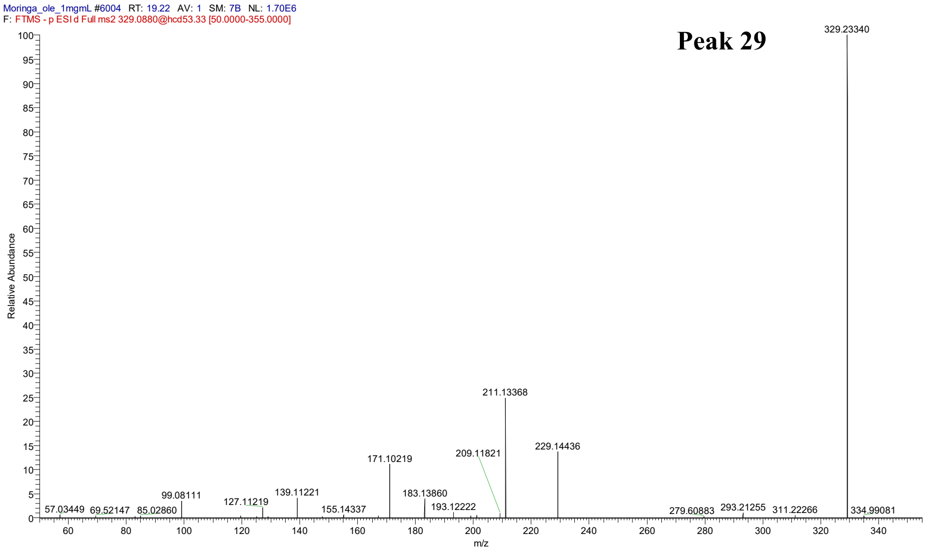

Fig. S11 HR-ESI-MS/MS of peaks **28** and **29**.

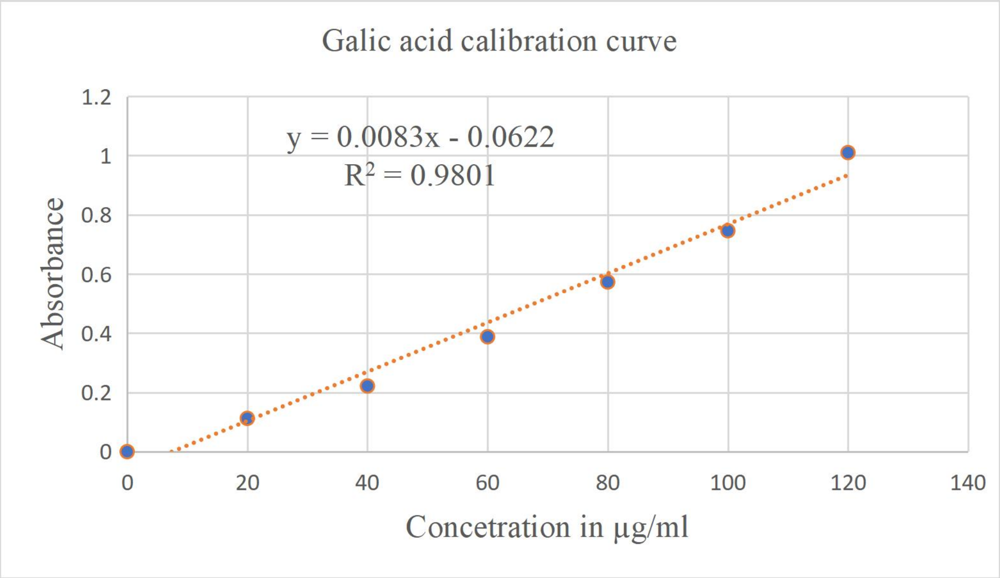

Fig. S12 Gallic acid calibration curve to determine total phenolic content.

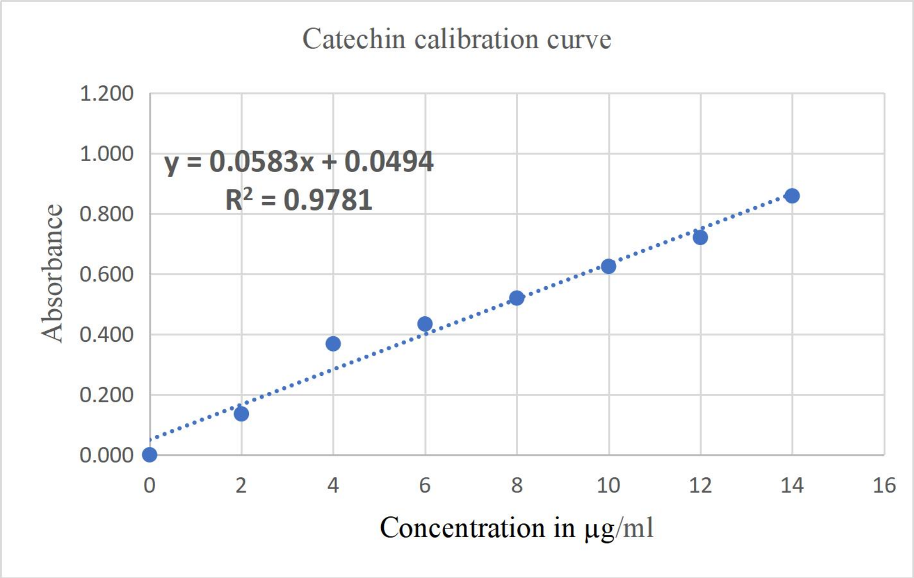

Fig. S13 Gallic acid calibration curve to determine total phenolic content.

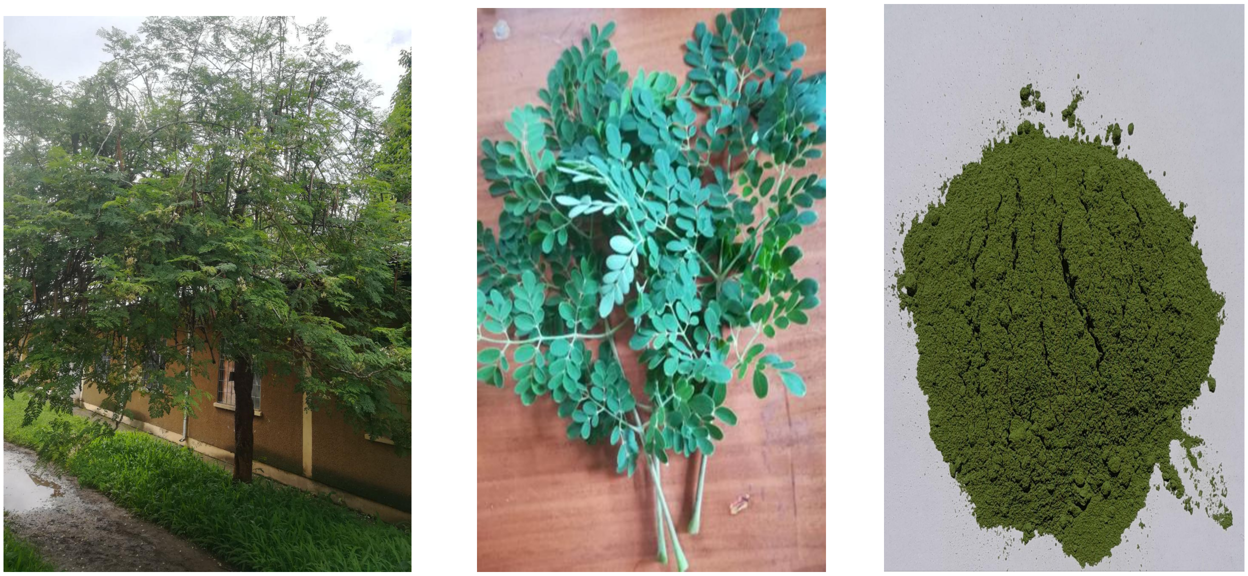

A. M. oleifera Tree B. *M. oleifera* leaves C. *M. oleifera* powder

Fig. S14 Parts of the studied *M. oleifera* species A) *M. oleifera* tree B) *M. oleifera* leaves C) M. *oleifera* powder

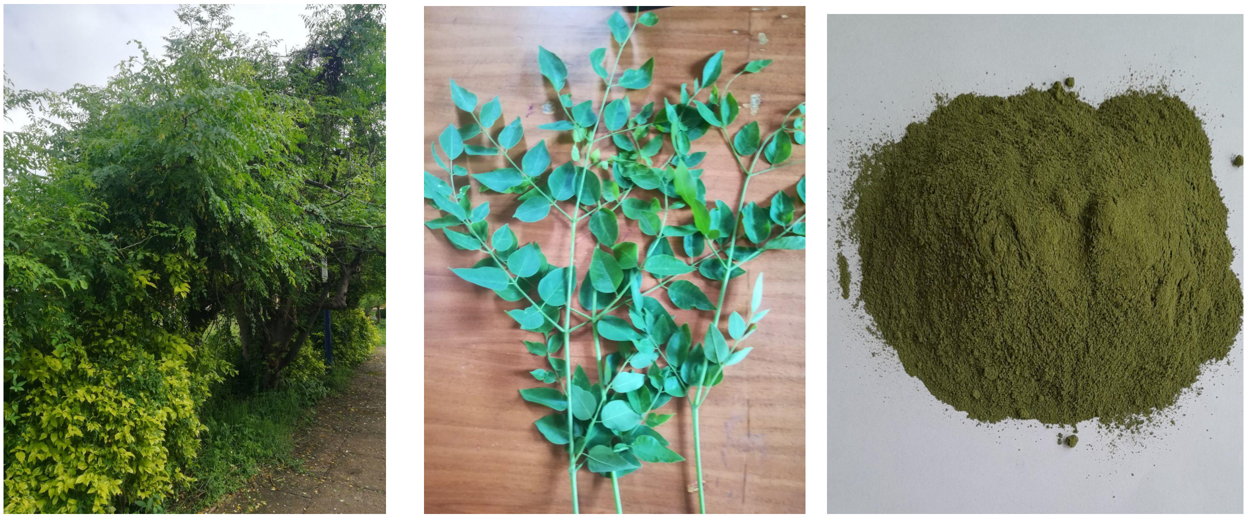

A. M. stenopetala Tree B. *M. stenopetala* leaves C. *M. stenopetala* powder

Fig. S15 Parts of the studied *M. stenopetala* species A) *M. stenopetala* tree B) *M. oleifera* leaves C) *M. stenopetala* powder

References

1 M. Vitiello, M. Pecoraro, M. De Leo, F. Camangi, V. Parisi, G. Donadio, A. Braca, S. Franceschelli and N. De Tommasi, *Antioxidants*, DOI:10.3390/antiox13010111.

2 Y. Asale, E. Dessalegn, D. Assefa and M. Abdisa, *Int. J. Food Prop.*, 2021, **24**, 354–363.

3 E. Dessalegn, *Ethiop. Pharm. J.*, 2016, **31**, 93.

4 C. Rodríguez-Pérez, R. Quirantes-Piné, A. Fernández-Gutiérrez and A. Segura-Carretero, *Ind. Crops Prod.*, 2015, **66**, 246–254.

5 H. Lin, H. Zhu, J. Tan, H. Wang, Z. Wang, P. Li, C. Zhao and J. Liu, *Molecules*, DOI:10.3390/molecules24050942.

6 F. Braham, L. M. P. F. Amaral, K. Biernacki, D. O. Carvalho, L. F. Guido, J. M. C. S. Magalhães, F. Zaidi, H. K. S. Souza and M. P. Gonçalves, *Foods*, DOI:10.3390/foods11172641.

7 K. Masike, M. I. Mhlongo, S. P. Mudau, O. Nobela, E. N. Ncube, F. Tugizimana, M. J. George and N. E. Madala, *Chem. Cent. J.*, 2017, **11**, 1–7.

8 E. N. Ncube, M. I. Mhlongo, L. A. Piater, P. A. Steenkamp, I. A. Dubery and N. E. Madala, *Chem. Cent. J.*, 2014, **8**, 1–10.

9 M. N. Clifford, J. Kirkpatrick, N. Kuhnert, H. Roozendaal and P. R. Salgado, *Food Chem.*, 2008, **106**, 379–385.

10 A. Chiș, P. A. Noubissi, O. L. Pop, C. I. Mureșan, M. A. Fokam Tagne, R. Kamgang, A. Fodor, A. V. Sitar-Tăut, A. Cozma, O. H. Orășan, S. C. Hegheș, R. Vulturar and R. Suharoschi, *Plants*, DOI:10.3390/plants13010020
